# A methylation‐driven gene panel predicts survival in patients with colon cancer

**DOI:** 10.1002/2211-5463.13242

**Published:** 2021-07-28

**Authors:** Yaojun Peng, Jing Zhao, Fan Yin, Gaowa Sharen, Qiyan Wu, Qi Chen, Xiaoxuan Sun, Juan Yang, Huan Wang, Dong Zhang

**Affiliations:** ^1^ Emergency Department The First Medical Center Chinese PLA General Hospital Beijing China; ^2^ College of Graduate Chinese PLA General Hospital Beijing China; ^3^ Department of Scientific Research Administration Chinese PLA General Hospital Beijing China; ^4^ Department of Oncology The Second Medical Center & National Clinical Research Center of Geriatric Disease Chinese PLA General Hospital Beijing China; ^5^ Department of Pathology The First Affiliated Hospital of Inner Mongolia Medical University Hohhot City China; ^6^ Department of Oncology The First Medical Center Chinese PLA General Hospital Beijing China; ^7^ Department of Traditional Chinese Medicine The First Medical Center Chinese PLA General Hospital Beijing China; ^8^ National Clinical Research Center for Cancer Key Laboratory of Cancer Prevention and Therapy Tianjin's Clinical Research Center for Cancer Tianjin Medical University Cancer Institute and Hospital China; ^9^ Department of Oncology Surgery Tianjin Cancer Hospital Airport Free Trade Zone Hospital China; ^10^ Department of Cardiothoracic Surgery Tianjin Fourth Center Hospital China

**Keywords:** colon cancer, DNA methylation, epigenetics, integrative analyses, prognosis, TCGA

## Abstract

The accumulation of various genetic and epigenetic changes in colonic epithelial cells has been identified as one of the fundamental processes that drive the initiation and progression of colorectal cancer (CRC). This study aimed to explore functional genes regulated by DNA methylation and their potential utilization as biomarkers for the prediction of CRC prognoses. Methylation‐driven genes (MDGs) were explored by applying the integrative analysis tool (methylmix) to The Cancer Genome Atlas CRC project. The prognostic MDG panel was identified by combining the Cox regression model with the least absolute shrinkage and selection operator regularization. Gene set enrichment analysis was used to determine the pathways associated with the six‐MDG panel. Cluster of differentiation 40 (*CD40*) expression and methylation in CRC samples were validated by using additional datasets from the Gene Expression Omnibus. Methylation‐specific PCR and bisulfite sequencing were used to confirm DNA methylation in CRC cell lines. A prognostic MDG panel consisting of six gene members was identified: *TMEM88*, *HOXB2*, *FGD1*, *TOGARAM1*, *ARHGDIB* and *CD40*. The high‐risk phenotype classified by the six‐MDG panel was associated with cancer‐related biological processes, including invasion and metastasis, angiogenesis and the tumor immune microenvironment. The prognostic value of the six‐MDG panel was found to be independent of tumor node metastasis stage and, in combination with tumor node metastasis stage and age, could help improve survival prediction. In addition, the expression of *CD40* was confirmed to be regulated by promoter region methylation in CRC samples and cell lines. The proposed six‐MDG panel represents a promising signature for estimating the prognosis of patients with CRC.

## Graphical abstract

By applying the methylmix algorithm to The Cancer Genome Atlas colon adenocarcinoma samples, we identified a prognostic methylation‐driven gene panel consisting of six genes (*TMEM88*, *HOXB2*, *FGD1*, *TOGARAM1*, *ARHGDIB* and *CD40*) that we have validated internally. We confirmed that *CD40*, a promising target for cancer immunotherapy, was regulated by methylation of its promoter region in colon cancer tissues and cell lines.

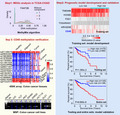

**Abbreviations**
450 K array: Illumina Human Methylation 450 Beadchip
5‐Aza: 5‐aza‐2′‐deoxycytidine
AIC: Akaike Information Criterion
ARHGDIB: Rho GDP dissociation inhibitor beta
AUC: area under the ROC curve
BSSQ: bisulfite sequencing
CD40: cluster of differentiation 40
CIMP: CpG island methylator phenotype
COAD: colon adenocarcinoma
CRC: colorectal cancer
FDR: false discovery rate
FGD1: FYVE, RhoGEF and PH domain containing 1
GEO: Gene Expression Omnibus
GSEA: gene set enrichment analysis
HOXB2: homeobox B2
KEGG: Kyoto Encyclopedia of Genes and Genomes
LASSO: least absolute shrinkage and selection operator
MDG: methylation‐driven gene
MSP: methylation‐specific PCR
OS: overall survival
ROC: receiver operating characteristic
SOCS126: suppressor of cytokine signaling 126
TCGA: The Cancer Genome Atlas
TMEM88: transmembrane protein 88
TNF: tumor necrosis factor
TNM: tumor node metastasis
TOGARAM1: TOG array regulator of axonemal microtubules 1
TSS: transcriptional start site

Colorectal cancer (CRC), which has heterogeneous outcomes and distinct underlying pathobiological and molecular features, ranks third in cancer incidence and second in cancer‐related mortality worldwide [1]. Generalized screening of high‐risk populations with precursor‐initiating adenomas at age 50 years or older is an effective and durable strategy to detect early‐stage cancers, reducing the incidence and mortality of CRC [2, 3, 4]. Surgical resection of the primary cancer and/or limited metastasis is the only approach for attempted cure, and additional chemoradiation may improve outcomes in some patients [4, 5]. However, relapse or metachronous metastasis occurs in a subset of these patients, leading to increased mortality [6]. Therefore, robust diagnostic, prognostic and predictive biomarkers are urgently needed.

Currently, the American Joint Committee on Cancer tumor node metastasis (TNM) staging system is the only well‐recognized stratification method used in clinical practice to guide therapeutic decisions and to predict the prognoses of patients with CRC [7, 8]. However, the fact that the survival times in patients with CRC with the same TNM stage often vary highlights the need for more accurate strategies [9]. It is widely known that genetic changes, such as gene mutations, contribute to cancer formation and can be used to predict the outcomes of patients with CRC [10, 11]. Recently, a consensus has been reached that epigenetic alterations, such as aberrant DNA methylation, abnormal histone modifications and altered expressions of noncoding RNA, occur early and manifest more frequently than genetic changes in CRC [12]. In addition, advances in genomic technology and bioinformatics have led to the identification of specific epigenetic alterations as potential clinical biomarkers in patients with CRC [12, 13]. For example, with the availability of genomic platforms capable of broadly surveying gene expression and DNA methylation, such as The Cancer Genome Atlas (TCGA) project, we can now identify genomic subtypes of CRC [14, 15], and the CpG island methylator phenotype (CIMP) has undoubtedly been one of the most promising epigenetic biomarkers for the prognostication of patients with CRC [12, 16].

By applying an integrative analysis tool (methylmix) to CRC samples from TCGA project, this study aimed to explore functional genes regulated by DNA methylation and the potential of these DNA methylation changes to become biomarkers for the prediction of CRC prognosis. We identified a prognostic methylation‐driven gene (MDG) panel consisting of six gene members: transmembrane protein 88 (*TMEM88*); homeobox B2 (*HOXB2*); FYVE, RhoGEF and PH domain containing 1 (*FGD1*); TOG array regulator of axonemal microtubules 1 (*TOGARAM1*); RhoGDP dissociation inhibitor beta (*ARHGDIB*); and cluster of differentiation 40 (*CD40*). The high‐risk phenotype classified by the six‐MDG panel was associated with cancer‐related biological processes, including invasion, metastasis, angiogenesis, tumor immune microenvironment, among others. We also confirmed the expression and methylation of *CD40*, a member of the six‐MDG panel, in CRC samples and cell lines.

## Materials and methods

### Data acquisition and preprocessing

The TCGA‐Assembler was used to download level 3 DNA methylation data from colon adenocarcinoma (COAD) samples, measured by the Illumina Human Methylation 450 Beadchip (450 K array), from the TCGA data portal (https://portal.gdc.cancer.gov/) [17]. These data were preprocessed via TCGA pipelines and presented in the form of beta (β)‐values, a ratio between methylated probe and total probe intensities. Probe‐level data were condensed to a summary β‐value by calculating the average methylation value for all CpG sites associated with a gene [18].

In total, 353 DNA methylation samples, including 315 COAD samples and 38 tumor‐adjacent samples, were obtained. Methylation data were normalized using the limma r package. Level 3 RNA sequencing data and clinical information were retrieved from the TCGA data portal. Of 521 transcriptome profiles, 41 cases were obtained from tumor‐adjacent tissues, while the remaining 480 cases were COAD tissues. The transcriptome data were normalized and log_2_ transformed with the functions of DEGList and calcNormFactors in the edger package [19]. The clinical data were preprocessed by exclusion of samples without survival status, and patients whose survival time was less than 30 days were also removed [20]. Two additional CRC profile datasets, GSE8671 and GSE42752, were downloaded from Gene Expression Omnibus (GEO; https://www.ncbi.nlm.nih.gov/geo/) and used to examine the expression and methylation of *CD40*, respectively. The GSE8671 dataset contains transcriptional data from 32 patients with COAD with adjacent normal mucosa, which was evaluated by Affymetrix Human Genome U133 Plus 2.0 Array [21]. The GSE42752 dataset includes a genome‐wide DNA methylation profile obtained from 22 COAD samples with corresponding adjacent normal colon mucosa and 20 samples from healthy colon mucosa using a 450 K array [22]. The earlier data are available for research with no restrictions, and this study was performed in accordance with the guidelines provided by TCGA and GEO.

### Identification of MDGs

To identify MDGs, we used the methylmix r package to perform an analysis integrating gene expression and DNA methylation data. In the methylmix algorithm, the methylation state of a gene is established by a β‐mixture model, and hypomethylated or hypermethylated genes are determined by comparing their differential methylation state in cancer versus normal tissues [false discovery rate (FDR) < 0.05] [23, 24]. To be functionally relevant, MDGs should have a significant predictive effect on gene expression, implying that methylation is inversely associated with transcription (Pearson’s coefficient < −0.3, *P* < 0.05) [23, 24].

### Construction of a prognostic model for survival prediction

Survival analysis was performed on 281 patients with COAD for whom both methylation and survival information [overall survival (OS) > 30 days] were available. First, we randomly designated 50% of the patients with COAD as the training set and the remaining 50% of patients with COAD as the testing set. Data matrices were generated by combining the methylation levels of the identified MDGs with corresponding follow‐up data from the patients with COAD. Then univariate Cox regression analysis was performed to screen for MDGs that were significantly associated with OS (*P* < 0.05) based on their methylation β‐value in the training set. Least absolute shrinkage and selection operator (LASSO) estimation, a well‐suited approach when there is a large number of correlated covariates for model construction in the patient cohort [25], was performed to penalize the effect of multicollinearity using the glmnet r package [26]. MDGs that survived the LASSO estimation were subsequently subjected to multivariate Cox regression to construct a best‐fitting prognostic model, with the Akaike Information Criterion (AIC) indicating model fitness [27]. The survival r package was used for the univariate and multivariate Cox regression analyses.

### Risk score calculation

The risk score was calculated by a linear combination of the methylation β‐value of the selected MDGs weighted by their estimated regression coefficient in the multivariable Cox regression analysis, as discussed previously [28]. Patients with COAD were classified into high‐ or low‐risk groups, using the median risk score of the training set as the cutoff value.

### Gene set enrichment analysis

Gene set enrichment analysis (GSEA) [29] was used to determine whether the members of a given gene set were generally associated with the risk score derived from the prognostic six‐MDG panel. The risk score (high or low) was designated as the phenotype, and the analysis was conducted using the matched gene expression profile. Random sample permutations and the significant threshold were set at 1000 times and FDR < 0.01, respectively. GSEA was performed using the JAVA program (http://software.broadinstitute.org/gsea/index.jsp) using the MSigDB C2 CP: Kyoto Encyclopedia of Genes and Genomes (KEGG) gene set collection. The enriched KEGG pathways were ranked by normalized enrichment score, and if a gene set had a positive normalized enrichment score, the high expression level of the majority of its members was positively related to the high‐risk score phenotype.

### Experimental validation of CRC cell lines

A panel of six CRC cell lines (RKO, SW480, SW620, HCT116, DLD1 and LoVo) was included in this study. All cell lines were preserved at our institute (The First Medical Center, Chinese PLA General Hospital, Beijing, China) and were cultured in Roswell Park Memorial Institute 1640 medium supplemented with 10% fetal bovine serum and 1% penicillin–streptomycin.

mRNA expression of *CD40* in CRC cell lines with or without 5‐aza‐2′‐deoxycytidine (5‐Aza; Sigma, St. Louis, MO, USA) treatment (2 μm for 96 h) was evaluated using semiquantitative RT‐PCR as previously described [30]. Genomic DNA was prepared using the Proteinase K method. Bisulfite treatment, methylation‐specific PCR (MSP) and bisulfite sequencing (BSSQ) were performed as previously described [31]. Genomic sequences around the transcriptional start site (TSS) were used as the template for CpG island prediction and the design of MSP and BSSQ primers using methyl primer express software v1.0 (Thermo Fisher Scientific, Waltham, MA, USA). The primers for RT‐PCR, MSP and BSSQ are listed in Table S1.

Total protein of *CD40* in these CRC cell lines was measured by western blotting, as previously described [30], using β‐actin as the loading control. The antibodies used for western blotting were purchased from Proteintech (Wuhan, China). We also examined the membrane expression of *CD40* using flow cytometry. Cells were harvested using trypsin and were washed with phosphate‐buffered solution before incubation with and without phycoerythrin‐tagged mouse monoclonal antibody to human *CD40* (Sino Biological, Beijing, China) at 4 °C for 30 min. Then the cells were washed twice to remove unbound antibodies before being measured on a FACSCalibur flow cytometer (BD Biosciences, Franklin Lakes, NJ, USA).

### Statistical analysis

The Mann–Whitney and Wilcoxon matched‐pairs signed‐rank tests were used to analyze the differences in DNA methylation, gene expression and risk score in nonpaired and paired samples, respectively. The relationship between the risk score and clinicopathological characteristics was analyzed using the Chi‐square or Fisher's exact test. Survival differences between the high‐ and low‐risk groups were evaluated using Kaplan–Meier analysis, and the log rank test was used as a statistical method. Multivariate Cox regression and data stratification analyses were performed to determine whether the risk score derived from the prognostic MDG panel was independent of the clinicopathological features of the patients with COAD. A receiver operating characteristic (ROC) curve was used, and the area under the ROC curve (AUC) was calculated to compare the sensitivity and specificity of survival prediction based on age, TNM stage, the risk score derived from the prognostic six‐MDG panel and a combination thereof. Statistical tests were conducted using prism8 (GraphPad Software, San Diego, CA, USA) or r 3.6.0 using the corresponding aforementioned r package.

## Results

### Screening MDGs in COAD

**Fig. 1:**
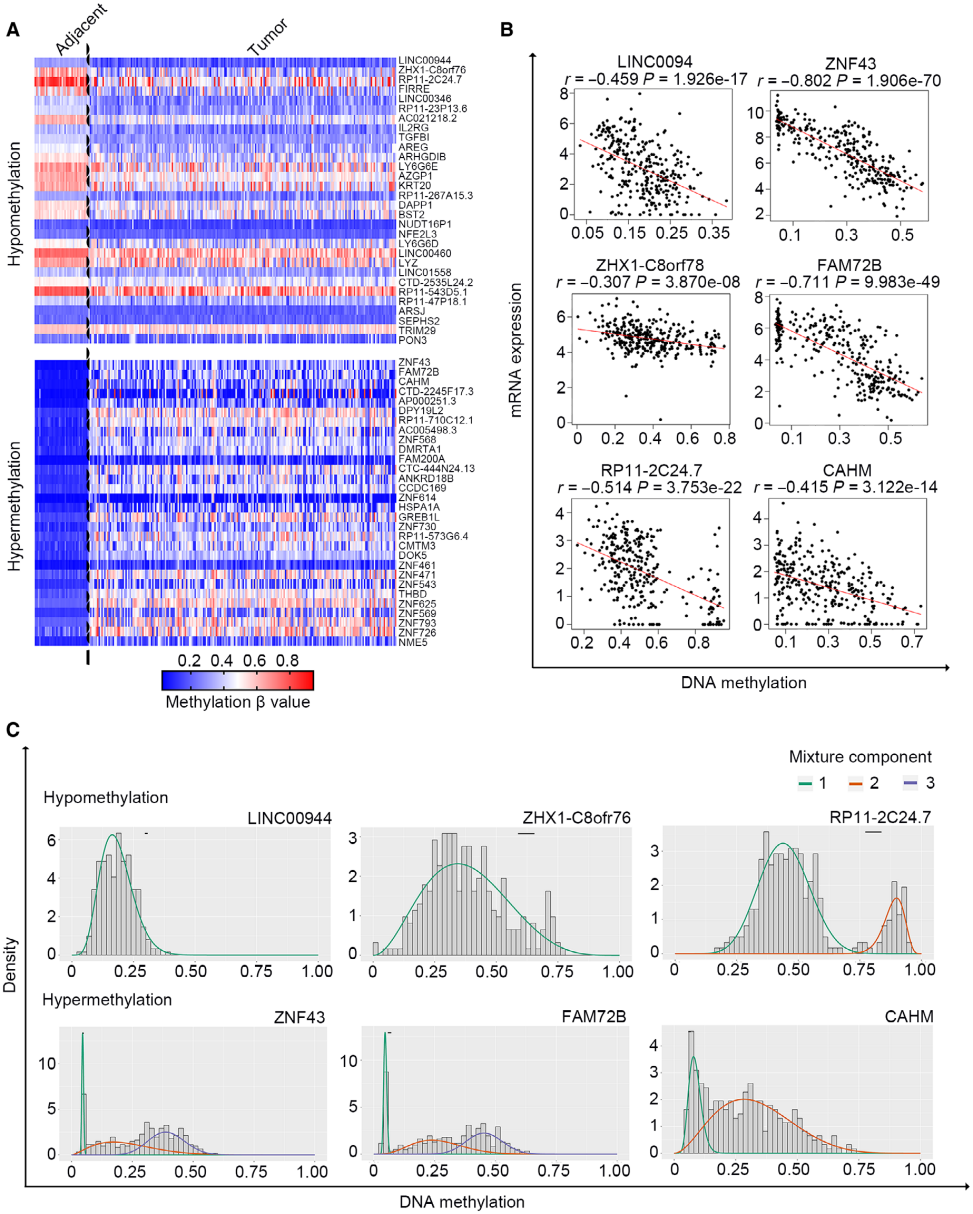
Screening for MDGs in CRC. (A) The methylation profile of the 30 most significant hypomethylated and hypermethylated MDGs in adjacent (*n* = 38) and CRC (*n* = 315) tissues. (B) The association between gene expression and DNA methylation of the top three hypomethylated and hypermethylated MDGs in CRC samples with both data available (*n* = 309). Pearson’s correlation analysis for each selected MDG was conducted (red line). (C) The mixture models of the top three hypomethylated and hypermethylated MDGs in adjacent (*n* = 38) and CRC samples (*n* = 315). The mixture components analyzed by the methylmix algorithm indicate the fitting curve of the distribution of the methylation values (β‐values) across all the samples (*n* = 353); the horizontal black bar represents the distribution of methylation values in the adjacent samples (*n* = 38).

We first prepared corresponding expression and methylation data, and three data matrices were acquired: a gene expression profile of 308 tumor tissues and two methylation profiles of 38 adjacent and 308 tumor tissues, respectively. These profiles were used as input data for the methylmix r package, with which differential and correlation analyses between DNA methylation and gene expression were conducted. Based on the screening criteria, a total of 299 MDGs were identified (Table S2). The methylation profiles of the most significant 30 hypomethylated and hypermethylated MDGs (ranked by the β‐value difference between tumor and adjacent tissues) are shown in Fig. 1A. The correlations between DNA methylation and gene expression and the methylation mixture models of the top three MDGs are shown in Fig. 1B,C, respectively.

### Identification of a prognostic six‐MDG panel from the training set

**Fig. 2:**
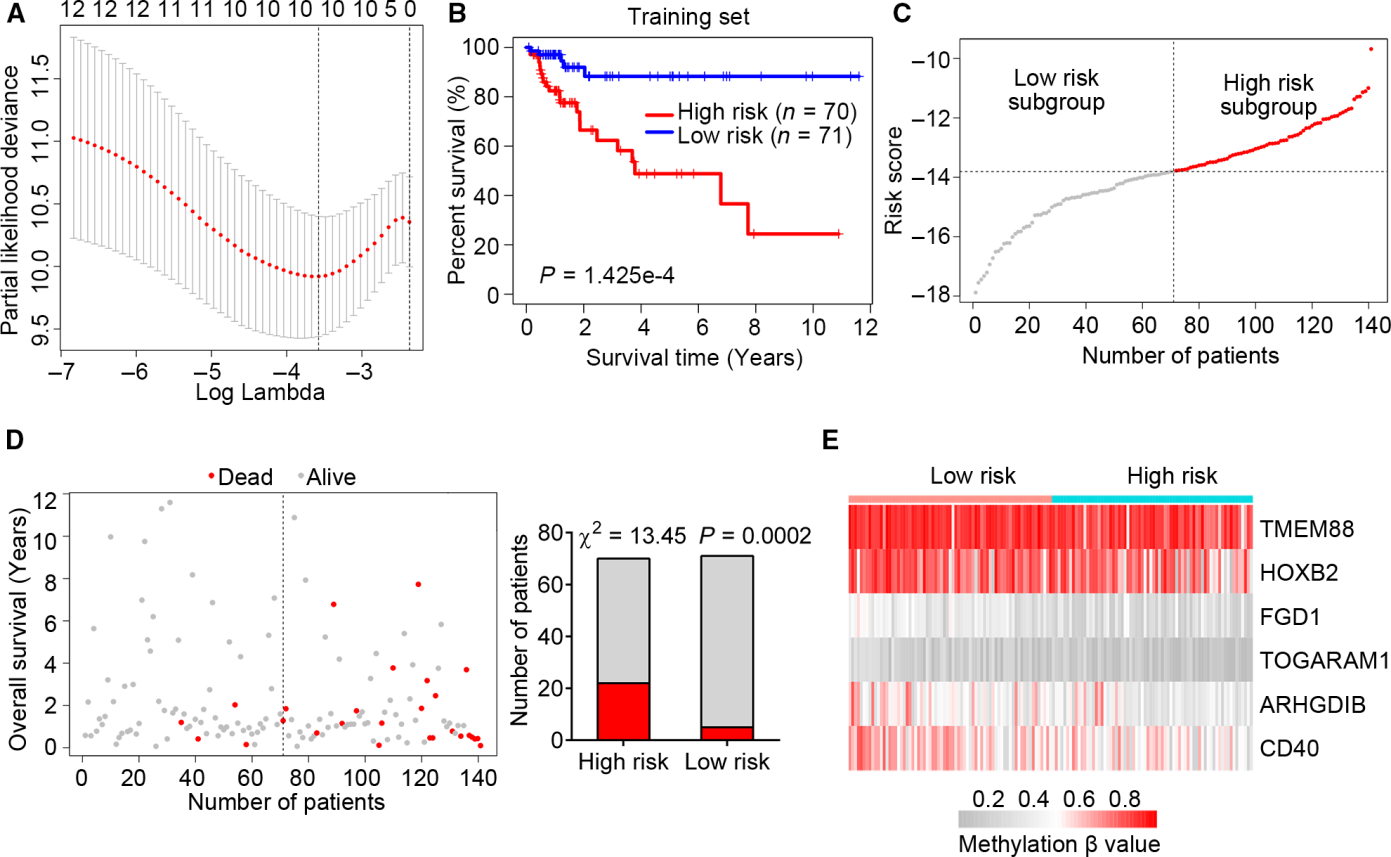
Identification of a prognostic six‐MDG panel in the training set. (A) Ten‐fold cross‐validation for tuning parameter selection in the LASSO model. The partial likelihood deviance corresponding to each lambda value was shown as mean ± SD. The dotted vertical line (left) indicates the optimal value by minimum criteria. (B) The Kaplan–Meier estimate of the OS using the six‐MDG panel in the training set. Patients with CRC were divided into high‐ (*n* = 70) and low‐risk (*n* = 71) subgroups based on the median risk score value. The difference between the two curves was determined by the two‐sided log rank test. (C) The distribution of risk scores derived from the six‐MDG panel applied to the training set. (D) The distribution of the survival status of patients with CRC in the training set. The difference between the high‐ and low‐risk subgroups was determined using the Chi‐square test. (E) The methylation profile of the six MDGs in the training set.

After preprocessing the methylation and clinical data, a total of 281 patients with COAD with adequate methylation and follow‐up data were included in the survival analysis. The clinical information for these 281 patients is summarized in Table S3. The patients were randomly split into a training set (*n* = 141) and testing set (*n* = 140). To identify certain prognostic MDGs, we performed univariate Cox regression analysis on the training set, and 12 prognosis‐related MDGs (*P* < 0.05; Table S4) were chosen for subsequent LASSO estimation. Ten MDGs survived the LASSO regularization (Fig. 2A) after penalization of the multicollinearity effect and were further subjected to multivariate Cox regression analysis to construct a best‐fitting prognostic model. The AIC was used to indicate the model fitness. Finally, a prognostic DNA methylation gene panel consisting of six MDGs (*TMEM88*, *HOXB2*, *FGD1*, *TOGARAM1*, *ARHGDIB* and *CD40*) was identified. Detailed information on the six MDGs is presented in Table 1. The methylation profile, correlations between gene expression and DNA methylation, and methylation mixture models of the six MDGs are shown in Fig. S1. The prognostic six‐MDG panel included one gene (*ARHGDIB*) with a statistically nonsignificant *P* value (*P* = 0.071; Table 1); however, this six‐MDG panel had the lowest AIC, representing the best model fitness, and the overall effect was significant (AIC = 202.86, global *P* [log rank] < 0.001).

**Table 1:** Six individual genes of the MDG panel associated with OS of patients with colon cancer. BC, bladder cancer; Chr, chromosome; ESCC, esophageal squamous cell carcinoma; HCC, hepatocellular carcinoma; LUSC, lung squamous cell carcinoma; NR, not reported; NSCLC, non‐small cell lung cancer; OC, ovarian cancer; OSCC, oral squamous cell carcinoma.

Gene symbol	Description	Chr	Coefficient	*P* value	Associated with DNA methylation in cancer
*TMEM88*	Transmembrane protein 88	17p13.1	−6.150	0.018	OC [52] and NSCLC [61]
*HOXB2*	Homeobox B2	17q21.32	−3.593	0.001	ESSC [62], OSCC [63] and BC [42]
*FGD1*	FYVE, RhoGEF and PH domain containing 1	Xp11.22	−7.287	0.003	HCC [64]
*TOGARAM1*	TOG array regulator of axonemal microtubules 1	14q21.2	−7.861	0.042	NR
*ARHGDIB*	Rho GDP dissociation inhibitor beta	12p12.3	−3.622	0.071	BC [65], LUSC [66] and OC [67]
*CD40*	Cluster of differentiation 40	20q13.12	−4.288	0.004	NR

Next, a risk score model for OS prediction was created based on the methylation β‐values of these six MDGs, as follows:

$$\begin{array}{cc} \text{\textbackslash{}hskip-1.7pcRisk}\;\text{score} & =(‐6.150\times \text{methylation}\;\beta \text{-value}\;\text{of}\;TMEM88)+(‐3.593\times \text{methylation}\;\beta \text{-value}\;\text{of}\;HOXB2)\\ & \;+(‐7.287\times \text{methylation}\;\beta \text{-value}\;\text{of}\;FGD1)+(‐7.861\times \text{methylation}\;\beta \text{-value}\;\text{of}\;TOGARAM1)\\ & \;+(‐3.622\times \text{methylation}\;\beta \text{-value}\;\text{of}\;ARHGDIB)+(‐4.288\times \text{methylation}\;\beta \text{-value}\;\text{of}\;CD40). \end{array}$$

We then calculated the risk score for each patient with COAD and classified them into high‐ or low‐risk subgroups using the median risk score of the patients in the training set as the cutoff value.

Kaplan–Meier survival curve analysis of the training set showed that patients with COAD in the high‐risk group had a significantly shorter median OS than those in the low‐risk group (log rank *P* < 0.001; Fig. 2B). We also profiled the distribution of risk score, survival status and methylation β‐values in the training set (Fig. 2C–E). The risk scores of the patients in the training set ranged from −17.883 to −9.677, with a median risk score of −13.807 (Fig. 2C). Moreover, there were more patients alive in the low‐risk than the high‐risk group (χ^2^ = 13.45, *P* = 0.0002; Fig. 2D). Interestingly, the methylation levels of all six MDGs were higher in low‐risk than high‐risk patients (Fig. 2E), indicating that hypermethylation of the six‐MDG panel is a favorable prognostic factor for patients with COAD.

### The six‐MDG panel is predictive of survival in the testing and total sets

**Fig. 3:**
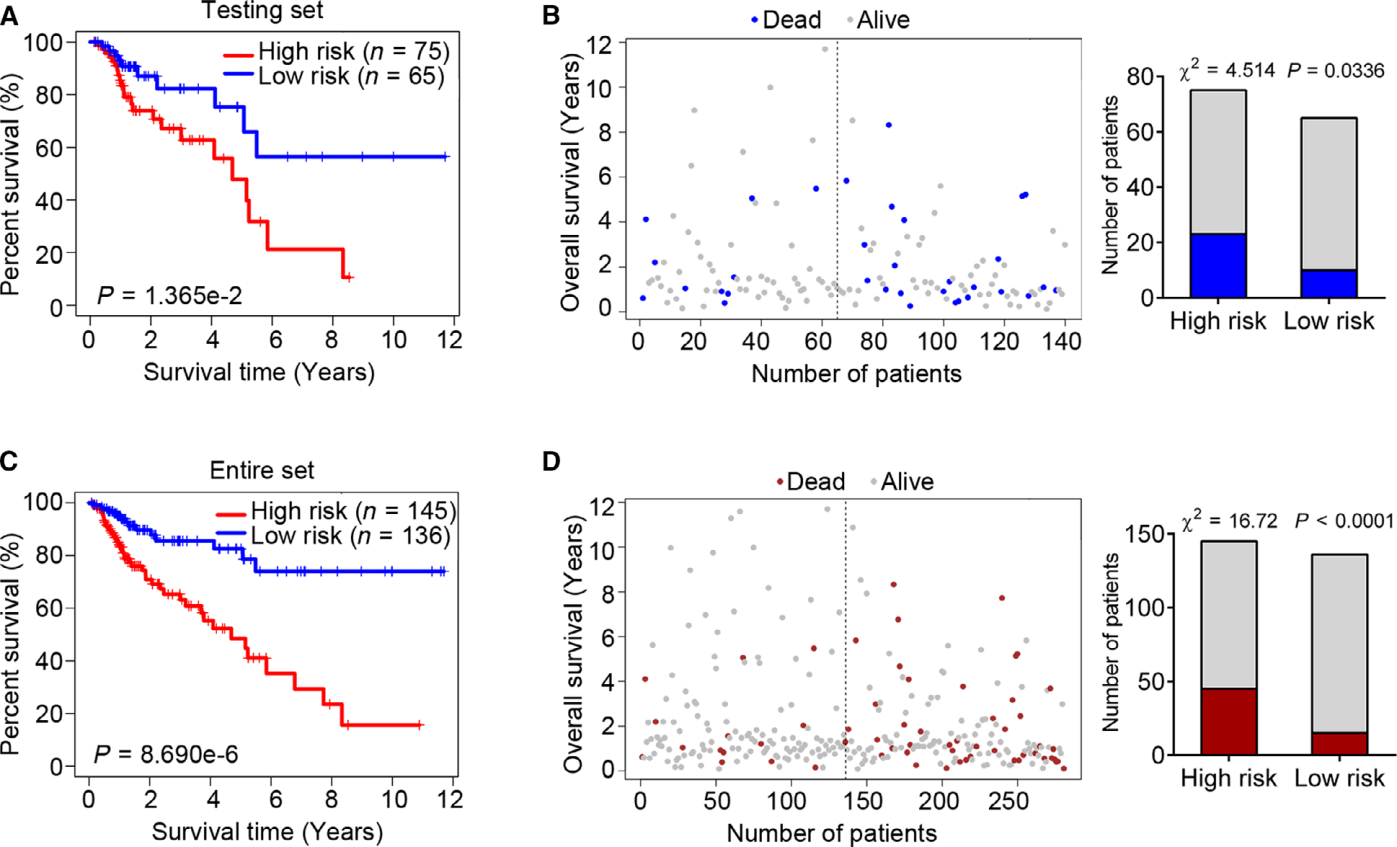
The six‐MDG panel is predictive of survival in the testing and total (training and testing) sets. (A) The Kaplan–Meier estimate of the OS using the six‐MDG panel in the testing set. Patients with CRC were divided into high‐ (*n* = 75) and low‐risk (*n* = 65) subgroups based on the median risk score of the training set. The difference between the two curves was determined by the two‐sided log rank test. (B) The distribution of survival status for the patients with CRC in the testing set. The difference between the high‐ and low‐risk subgroups was determined using the Chi‐square test. (C) The Kaplan–Meier estimate of the OS using the six‐MDG panel in the total set. (D) The distribution of survival status for the patients with CRC in the total set.

To further test the significance of the prognostic six‐MDG panel in patients with COAD, we used the testing and total sets as validation groups. Using the same risk score cutoff value obtained from the training set, we divided the patients with COAD in the testing set into high‐ (*n* = 75) and low‐risk (*n* = 65) groups. The results of Kaplan–Meier analysis demonstrated that the patients with COAD in the high‐risk group had a lower OS than those in the low‐risk group (log rank *P* = 0.0137; Fig. 3A), and that there were more patients in the low‐risk group than in the high‐risk group (χ^2^ = 4.514, *P* = 0.0336; Fig. 3B). We also performed the same analysis on the total set (training set plus testing set, *n* = 281), and the results were consistent with those of the training and testing sets individually (Fig. 3C,D). These results suggest that the selected six‐MDG panel can predict survival in both the training and total sets.

### The prognostic value of the six‐MDG panel is independent of TNM stage

**Fig. 4:**
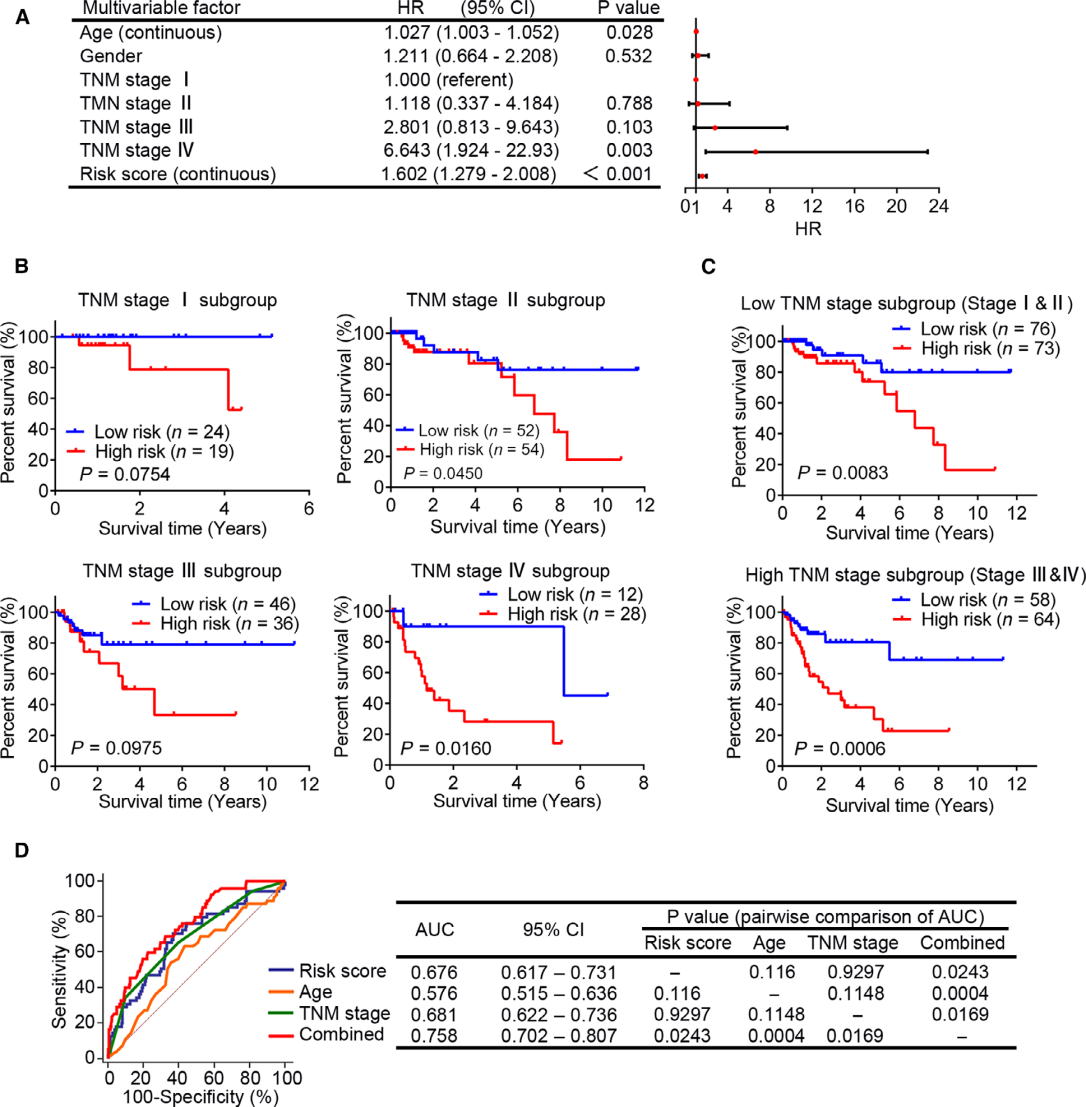
The prognostic value of the six‐MDG panel is independent of TNM stage. (A) The multivariate Cox regression analysis performed on 271 patients with CRC, using age, sex, TNM stage and risk score as covariates. Risk score and age were evaluated as continuous variables, and sex and TNM stage were evaluated as category variables. Red solid dots represent the hazard ratio (HR) of death, and open‐ended horizontal lines represent the 95% confidence intervals (CIs). All *P* values were calculated using Cox proportional hazards analysis. (B) The Kaplan–Meier curves for patients with CRC with TNM stages I (*n* = 43; upper left panel), II (*n* = 106; upper right panel), III (*n* = 82; bottom left panel) and IV (*n* = 40; bottom right panel). The difference between the two curves was determined by the two‐sided log rank test. (C) The Kaplan–Meier curves for patients with low (stages I and II, *n* = 149; upper panel) and high TNM stages (stages III and IV, *n* = 122; bottom panel). (D) ROC analysis of the sensitivity and specificity of OS prediction by age, TNM stage, risk score derived from the six‐MDG panel and combination of these three factors. *P* values were obtained from the pairwise comparisons of the AUCs.

TNM staging is a widely used and clinically useful classification system and is highly associated with the 5‐year OS in CRC [32]. Therefore, we aimed to clarify whether the prognostic value of the six‐MDG panel is independent of the TNM stage. We performed multivariate Cox regression and stratification analyses on the total set. After the exclusion of 10 patients who lacked adequate TNM staging information, we conducted multivariate Cox regression analysis on a total of 271 patients, with age, sex, TNM stage and risk score as covariates. The results showed that age, TNM stage and risk score remained independent prognostic factors (Fig. 4A). We then preformed data stratification analysis, with the patients stratified into four subgroups (stages I, II, III and IV). The results of the stratification analysis showed that the prognostic six‐MDG panel could identify patients with different OSs in the TNM stage II (log rank *P* = 0.0450) and IV (log rank *P* = 0.0160) subgroups (Fig. 4B), but was unable to sufficiently clarify the patients in the TNM stage I (log rank *P* = 0.0750) and TNM stage III (log rank *P* = 0.0975) subgroups with significantly disparate survival (Fig. 4B). This may be attributed to the small sample size or truncated dataset. Therefore, we combined low (stage I plus II) and high TNM stages (stage III plus IV) and found that the risk score could significantly identify patients with different prognoses in these two subgroups (log rank *P* = 0.0083 and 0.0006, respectively; Fig. 4C). These results suggest that the prognostic value of the six‐MDG panel is independent of the TNM stage.

Moreover, we performed ROC analysis to compare the sensitivity and specificity of OS prediction between the prognostic factors, including age, TNM stage, risk score derived from the six‐MDG panel and a combination of these three factors. As shown in Fig. 4D, there was no significant difference when the AUCs of the three prognostic factors (age, TNM stage and risk score) alone were compared pairwise (all *P* > 0.05). However, when these three prognostic factors were combined, the AUC was significantly greater than that of each prognostic factor alone (all *P* < 0.05). These results indicate that the combination of the three prognostic factors (age, TNM stage and risk score) may help improve survival prediction in patients with COAD.

### Assessment of biological pathways associated with the six‐MDG panel

**Fig. 5:**
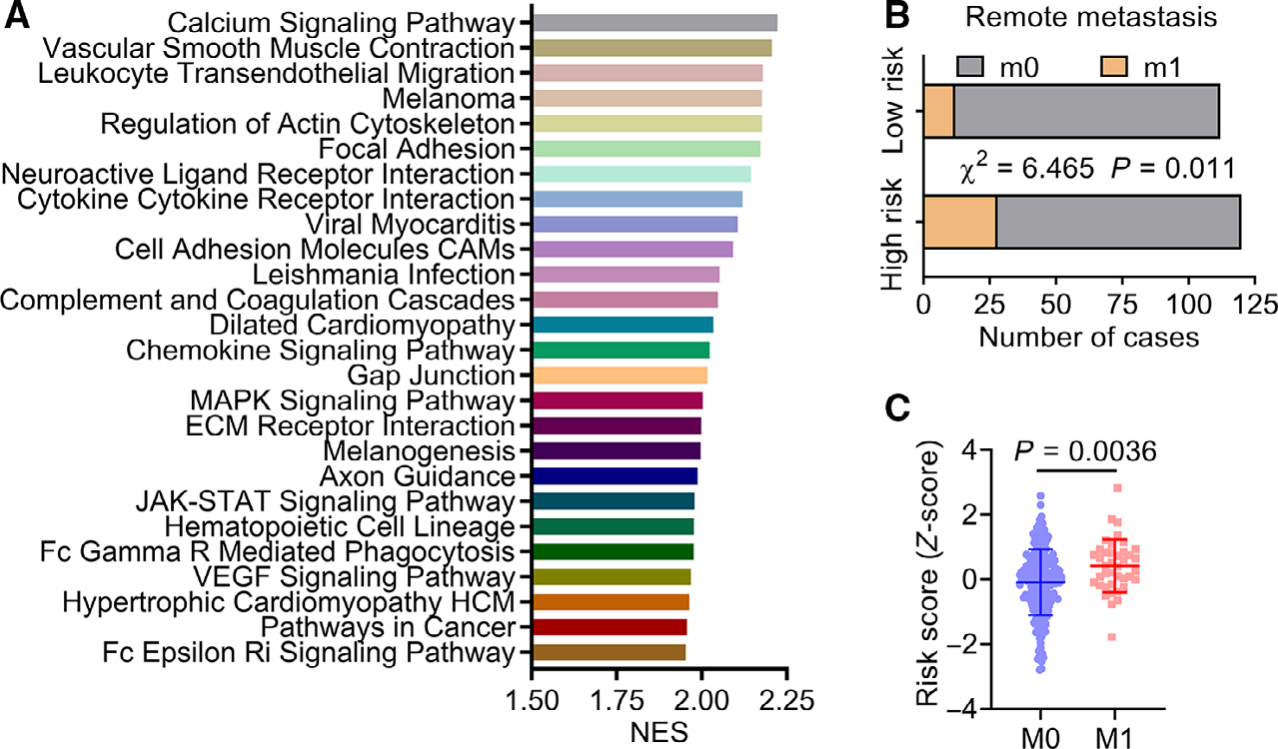
Assessment of relevant pathways and biological processes of the six‐MDG panel. (A) GSEA analysis showed significantly enriched KEGG pathways in CRC tissues with high‐risk phenotype (FDR < 0.01). (B) The relationship between remote metastasis and the risk score derived from the six‐MDG panel in patients with colon cancer (*n* = 232), the statistical difference of which was determined using the Chi‐square test. (C) Scatterplot of risk score for patients with (*n* = 40) or without (*n* = 192) metastasis. The Mann–Whitney test was used to determine the significance of the comparison.

We performed GSEA to identify relevant pathways that the six‐MDG panel may be involved in, using the risk score for phenotype classification. Gene sets significantly enriched (FDR < 0.01) for the high‐risk phenotype are shown in Fig. 5A. High‐risk scores were positively associated with the up‐regulation of several cancer‐related pathways, including invasion, metastasis, angiogenesis and tumor immune microenvironment. Vascular endothelial growth factor, for instance, a key regulator in the growth and maintenance of blood vessels, can directly modulate the vascular wall by loosening cell–cell contacts and increasing the permeability of blood vessels, which aids in the dissemination of tumor cells [33].

Next, we analyzed the relationship between clinicopathological features and the risk score derived from the six‐MDG panel in patients with COAD (Table 2). Consistent with the pathway analysis, the results showed that patients with COAD in the high‐risk group were more likely to have remote metastasis (χ^2^ = 6.465, *P* = 0.011; Table 2 and Fig. 5B). We also evaluated the risk score as a continuous variable and found that patients with metastasis tended to have higher risk scores than those without metastasis (*P* = 0.0036; Fig. 5C). Collectively, these results suggest that the selected six‐MDG panel is associated with cancer‐related signaling pathways and acts as an indicator of tumor metastasis.

**Table 2:** Correlations between clinicopathological features and risk scores derived from the six‐MDG panel. CEA, carcinoembryonic antigen.

Variable	*N*	High risk	Low risk	*P* value
Age (years)	281			0.535
≥60	185	93	92	
<60	96	52	44	
Sex	281			0.002*
Male	153	92	61	
Female	128	53	75	
History of colon polyps	213			0.304
Yes	50	21	29	
No	163	82	81	
Pretreatment CEA level (ng·μL^−1^)	184			0.067
≥5.0	61	38	23	
<5.0	123	59	64	
T stage	281			0.236
T3 + T4	231	123	108	
T1 + T2	50	22	28	
N stage	281			0.886
N1 + N2	119	62	57	
N0	162	83	79	
M stage	232			0.011*
M1	40	28	12	
M0	192	92	100	
TNM stage	271			0.570
Ⅰ + Ⅱ	122	64	58	
Ⅲ + Ⅳ	149	73	76	
Venous invasion	243			0.267
Yes	58	34	24	
No	185	93	92	
Tumor location	262			0.322
Right colon	164	80	84	
Left colon	98	54	44	

* *P* < 0.05.

### *CD40* is universally hypermethylated in CRC tissues

**Fig. 6:**
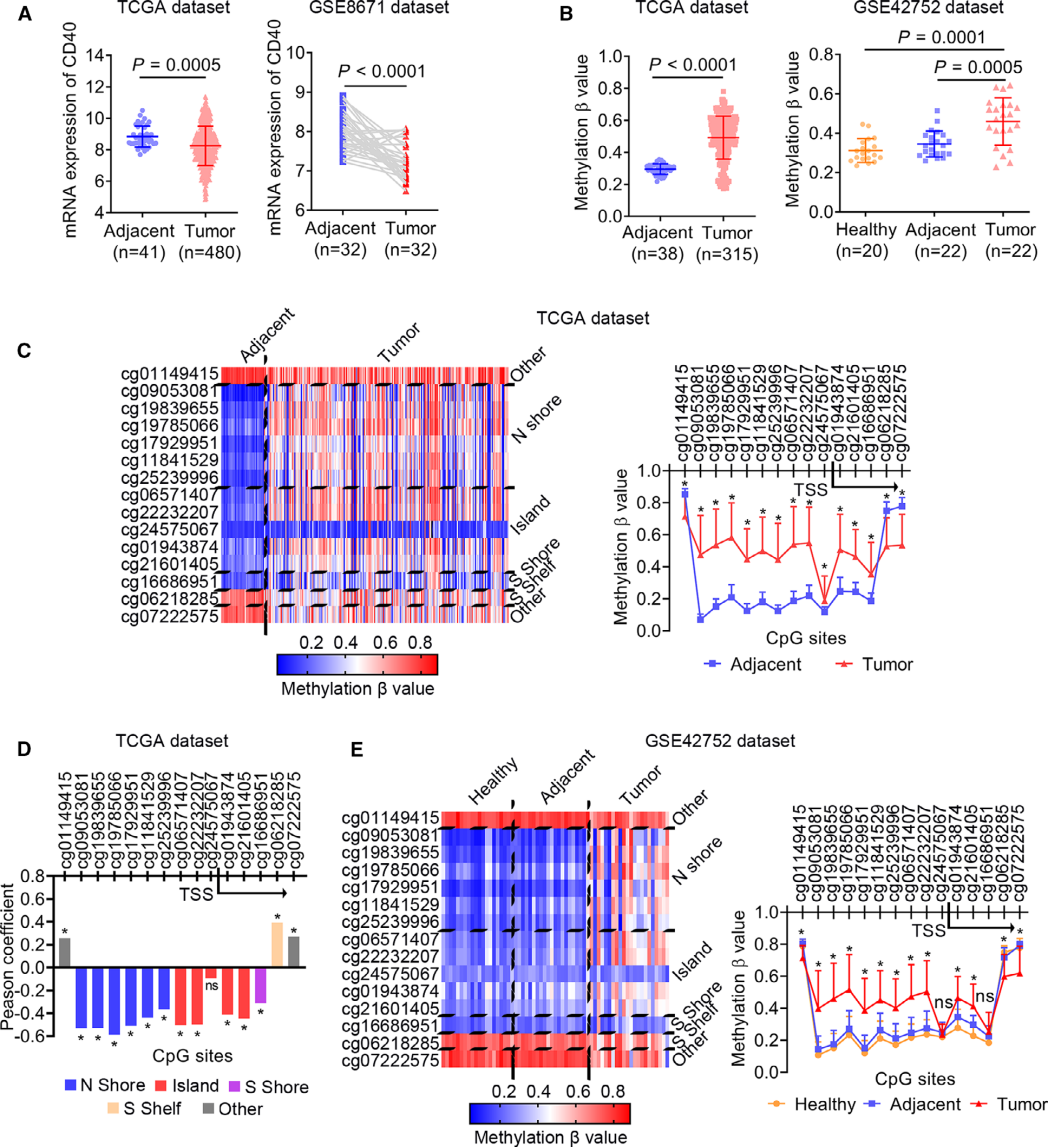
*CD40* is universally hypermethylated in CRC tissues. (A) Scatterplots of *CD40* mRNA expression between CRC and adjacent tissues from TCGA and GSE8671 dataset. The Mann–Whitney test was used to determine the significance of the comparison. (B) Scatterplots of *CD40* DNA methylation (β‐value) between CRC and adjacent tissues from TCGA dataset and among healthy, CRC and adjacent tissues from GSE42752 dataset. The Mann–Whitney and Wilcoxon matched‐pairs signed‐rank tests were used to analyze the differences between nonpaired and paired samples, respectively. (C) The methylation profile for all of the CpGs (*n* = 15) of *CD40* in CRC samples from TCGA dataset. The differences in CpG sites' methylation levels between tumor and adjacent tissues were determined by the Mann–Whitney test. (D) The Pearson’s coefficient correlations between *CD40* mRNA expression and methylation levels of all 15 CpG sites. (E) The methylation profile for all of the CpGs (*n* = 15) of *CD40* in CRC samples from the GSE42752 dataset. The differences in CpG sites' methylation levels between tumor and adjacent tissues were determined using the Mann–Whitney test. *P < 0.05. ns, no significance.

*CD40* is a member of the tumor necrosis factor (TNF) family and is a new immune‐modulating target with great potential in cancer treatment [34]. The regulation of *CD40* expression by DNA methylation has yet to be reported in the current literature and therefore deserves further investigation. We first examined the expression of *CD40* in patients with CRC from the TCGA and GSE8671 datasets. The transcriptional expression of *CD40* was significantly down‐regulated in CRC tissues compared with the healthy colon mucosa in both datasets (Fig. 6A). Next, we analyzed the overall methylation level of *CD40* in the TCGA and GSE42725 datasets, the results of which showed that *CD40* was hypermethylated in CRC tissues compared with adjacent and/or healthy colon mucosa in the two datasets (Fig. 6B). We also observed a negative correlation between mRNA expression and overall DNA methylation level in patients with COAD from TCGA dataset (Pearson’s *r* = −0.511, *P* < 0.001; Fig. S1B).

In addition, we analyzed the CpG site‐specific methylation status of all 15 CpG sites of *CD40*, assessed by the 450 K array. The CpG sites located in or near the CpG island (island, N shore and S shore) covering the TSS of *CD40* (12 CpG sites) were significantly hypermethylated in CRC tissues compared with the adjacent mucosa (Fig. 6C), and except for cg24575067, their methylation levels were negatively correlated with *CD40* expression (Fig. 6D). Interestingly, we observed a similar CpG site‐specific methylation pattern of *CD40* in the GSE42725 dataset (Fig. 6E). These results suggest that *CD40* is universally hypermethylated in CRC tissues, which may contribute to its transcriptional silencing.

### The expression of *CD40* is regulated by promoter methylation in CRC cell lines

**Fig. 7:**
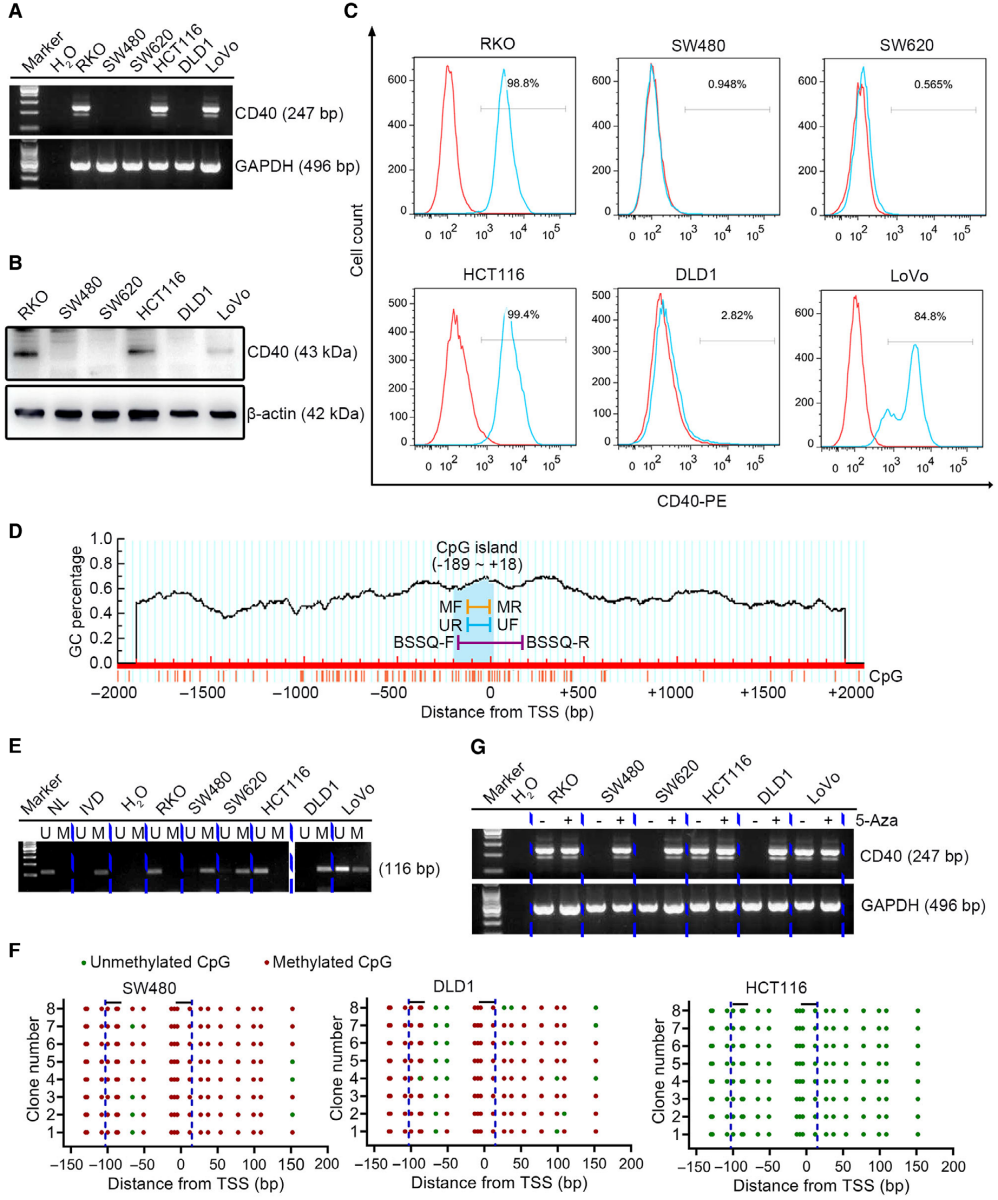
The expression of *CD40* is regulated by promoter methylation in CRC cell lines. (A) mRNA (247 bp), (B) total protein (43 kDa) and (C) membrane expression of *CD40* in six CRC cell lines (RKO, SW480, SW620, HCT116, DLD1 and LoVo). GAPDH (496 bp) and β‐actin (42 kDa) were used as the loading controls. (D) Schematic diagram of a CpG island in the promoter region of *CD40*. (E) Methylation status of *CD40* (116 bp) detected by MSP in CRC cell lines. (F) BSSQ of *CD40* performed in SW480, DLD1 and HCT116 cell lines. Red solid dots represent methylated CpG sites, and green solid dots denote unmethylated CpG sites. The horizontal black bar demarcates the primers of MSP, which are included in the region of BSSQ. (G) mRNA expression of *CD40* (247 bp) with (+) or without (−) treatment of 5‐Aza. GAPDH (496 bp) was used as the loading control. BSSQ‐F, bisulfite sequencing forward primer; BSSQ‐R, bisulfite sequencing reverse primer; IVD, *in vitro* methylated DNA; M, methylated alleles; MF, methylation forward primer; MR, methylation reverse primer; NL, normal lymphocyte DNA; UF, unmethylation forward primer; U, unmethylated alleles; UR, unmethylation reverse primer.

To better understand the regulation of *CD40* expression in CRC, we detected the levels of *CD40* expression in a panel of CRC cell lines, the results of which indicated that *CD40* mRNA expression was silenced in three of the six CRC cell lines (Fig. 7A). We confirmed the expression of *CD40* by performing western blot and flow cytometry analyses on the total and membrane protein levels of these six cell lines (Fig. 7B,C). Next, MSP and BSSQ were used to evaluate the methylation status of the *CD40* promoter region in these cell lines. The CpG islands situated in the *CD40* gene promoter region and the designed MSP and BSSQ primers are shown in Fig. 7D. MSP analysis revealed *CD40* promoter methylation in the three cell lines with silenced *CD40* expression (SW480, SW620 and DLD1) (Fig. 7E). BSSQ analysis of 19 CpG sites around the TSS showed dense methylation of the cell lines with silenced *CD40* expression that were examined (SW480 and DLD1), but not in the *CD40*‐expressing HCT116 cells (Fig. 7F). To test whether promoter methylation directly contributes to the transcriptional silencing of *CD40*, these six CRC cell lines were treated with 5‐Aza, a demethylation reagent. Restoration of *CD40* expression was induced using 5‐Aza in the three CRC cell lines with silenced *CD40* expression (Fig. 7G). These results indicate that *CD40* is silenced in CRC cell lines by promoter region hypermethylation.

## Discussion

Aberrant epigenetic changes drive carcinogenesis and subsequent tumor progression [35]. Of the various epigenetic modifications, DNA methylation is the key factor and is classically responsible for transcriptional silencing via the hypermethylation of CpG islands located in the promoter regions of tumor suppressor genes [36]. In addition, DNA hypomethylation has been implicated in the regulation of genome rearrangement and chromosomal instability, which may also contribute to carcinogenesis [36]. A plethora of gene‐specific studies have demonstrated that gene hypermethylation or hypomethylation can be used as an epigenetic biomarker to predict the behavior and prognosis of CRC [37]. There is also evidence of an association between the aberrant methylation of multiple genes and increased CRC aggressiveness [38]. For instance, Weisenberger *et al*. [] introduced the prevailing method used to identify CIMP in CRC, which is based on the methylation status of five genes: calcium voltage‐gated channel subunit alpha‐1 G (*CACNA1G*), insulin‐like growth factor 2 (*IGF2*), Neurogenin 1 (*NEUROG1*), runt‐related transcription factor 3 (*RUNX3*) and suppressor of cytokine signaling 1 (SOCS1). CIMP‐positive tumors were found to exhibit unique clinicopathological and molecular features, correlating with an overall unfavorable prognosis [39].

The advancement and prevalence of high‐throughput DNA methylation arrays have confirmed previously identified epigenetic changes and have also uncovered many new alterations, creating an opportunity to discover novel cancer‐related epigenetic biomarkers. By applying an integrative analysis tool to TCGA project, we aimed to explore key genes regulated by DNA methylation and their potential use as prognostic biomarkers of CRC. A model‐based algorithm (methylmix) was used to identify MDGs, from which we developed a prognostic MDG panel consisting of six genes (*TMEM88*, *HOXB2*, *FGD1*, *TOGARAM1*, *ARHGDIB* and *CD40*) in the training set (50% of the TCGA cohort). The six‐MDG panel exhibited favorable performance in OS prediction, which was validated through the test set (the remaining 50% of the TCGA cohort) and the total set (training and test sets). Multivariate Cox regression and data stratification analyses demonstrated that the prognostic value of the risk score derived from the six‐MDG panel was independent of the TNM stage. Furthermore, through ROC curve analysis, we found that the combination of age, TNM stage and the six‐MDG panel, the three independent prognostic factors revealed by the multivariate Cox regression analysis, may improve prognostication.

These six prognostic MDGs have different methylation values in tumors and their adjacent tissues, and their DNA methylation and mRNA expression levels are inversely correlated, indicating their potential roles in CRC. The GSEA pathway analysis we performed provided evidence that the six MDGs are involved in cancer‐related biological processes, including invasion, metastasis, angiogenesis, tumor immune microenvironment, among others. Up‐regulation of *HOXB2* was found to be an adverse prognostic indicator for stage I lung adenocarcinoma, promoting invasion by transcriptional regulation of metastasis‐related genes [40, 41]. In this study, *HOXB2* expression was negatively correlated with DNA methylation in CRC, and hypermethylation of *HOXB2* was associated with prolonged OS. However, Marsit *et al*. [42] revealed that increased promoter methylation of *HOXB2* in bladder cancer is significantly and independently associated with increased cancer aggressiveness. Further studies are needed to clarify the functional role of *HOXB2* in cancer. *ARHGDIB* has been identified as a regulator of tumor metastasis, but its role in cancer remains unknown [43]. *ARHGDIB* has been found to function as a positive regulator of cancer progression in ovarian [44], breast [45], colorectal [43] and gastric cancers [46], and as a negative regulator in Hodgkin's lymphoma [47], bladder cancer [48, 49] and lung cancer [50]. In this study, as in previous studies involving CRC, we found hypermethylation of *ARHGDIB* to be a favorable prognostic factor. *TMEM88* is a transmembrane protein that functions as an inhibitor of Wnt signaling [51], and *TMEM88* promoter hypomethylation is associated with platinum resistance in ovarian cancer [52]. The results of this study demonstrated that *TMEM88* is hypomethylated in the high‐risk group, which is associated with shorter OS in CRC. Therefore, we hypothesized that *TMEM88* may modulate the prognosis of CRC by altering the sensitivity of cancer cells to chemotherapy through mediation of promoter methylation, although further investigation is needed to confirm this. Ayala *et al*. [53] revealed that *FGD1* is central in the regulation of focal degradation of the extracellular matrices in invadopodia. They also demonstrated that *FGD1* is highly expressed in prostate and breast cancers, potentially leading to aberrant growth, invasiveness and/or metastasis [53]. *TOGARAM1* encodes a TOG domain array‐containing protein that regulates the structure of cilia microtubules [54]. The regulation of *TOGARAM1* expression by DNA methylation and its role in cancer have not yet been reported. *CD40* belongs to the TNF receptor family and is crucial to the mediation of a variety of immune and inflammatory responses [55]. *CD40* ligation provides essential activation signals for immune cells [55], although its function in the promotion or inhibition of tumorigenesis and progression via regulation of TNF alpha (TNFα)‐induced apoptosis [56], angiogenesis [57], tumor cell migration and invasion [58], and chemoresistance [59] is unknown. Agonist *CD40* antibodies have been developed and tested in clinical trials, in which impressive results have been noted, especially in pancreatic cancer [60]. We confirmed that the expression of *CD40* is regulated by promoter region hypermethylation in CRC tissues and cell lines, which may provide new insights into the combination of epigenetic therapy and *CD40*‐stimulating immunotherapy. Further investigation is needed to clarify the underlying mechanisms that potentiate MDGs as DNA methylation biomarkers for CRC.

This study had several limitations. First, no external validation was performed. We attempted to search for CRC cohorts with both methylation and follow‐up data in multiple cancer databases, including GEO and the International Cancer Genome Consortium project, among others, but no relevant available datasets were found. However, considering the number of patients included in the processes of model construction and internal validation for this study, the identified prognostic signature is unlikely to be random noise of the methylome. Second, experimental information regarding the regulatory mechanisms of all six prognostic MDGs on the methylation signature was presented. Third, the specific functional role of these prognostic MDGs in CRC remains unexplored.

## Conclusions

In summary, we identified an MDG‐related signature that acts as an independent prognostic factor in CRC, and its combination with clinical characteristics, including age and TNM stage, could help improve prognostication. Our results also confirmed that *CD40*, a member of the prognostic six‐MDG panel, is regulated by DNA methylation in CRC samples and cell lines. More testing is needed to obtain a complete picture of the regulatory mechanisms and functional roles of all six MDGs in CRC. In addition, clinical investigations of additional CRC patient cohorts are needed to validate our findings and to elaborate on their potential clinical utilization.

## Conflict of interest

The authors declare no conflict of interest.

## Author contributions

All authors contributed to the experimental design and data analysis of this study. YP, JZ, FY, GS and QW downloaded, organized and analyzed the data. YP, XS, QC and JY performed validation experiments in colon cell lines and drafted the manuscript. DZ and HW supervised the study and revised the manuscript. All authors read and commented on the manuscript and approved the final version.

## Supporting information

**Fig. S1.** The prognostic 6‐MDG panel in colon cancer. (A) The methylation profile of the six MDGs in the adjacent (n = 38) and CRC (n = 315) samples. (B) The associations between gene expression and DNA methylation of the six MDGs in CRC samples with both data available (n = 309). Pearson correlation analysis for each MDGs was conducted (red line). (C) The mixture models of the six MDGs in the adjacent (n = 38) and CRC (n = 315) samples. The mixture components analyzed by the MethylMix algorithm indicate the fitting curve of the distribution of methylation values (beta (β)‐values) across all samples (n = 353), and the horizontal black bar represents the distribution of methylation values in the adjacent samples (n = 38).

**Table S1.** Primers used in this study.

**Table S2.** Methylation‐driven genes identified in colon cancer patients.

**Table S3.** Clinical characteristics of 281 colon cancer patients included in survival analysis.

**Table S4.** 12 methylation‐driven genes significantly associated with overall survival of colon cancer patients screened by univariate Cox regression analysis in the training set (n = 141).

## Data Availability

Clinical information, high‐throughput sequencing counts and DNA methylation data were retrieved from TCGA data portal (https://portal.gdc.cancer.gov/) and from GEO (https://www.ncbi.nlm.nih.gov/geo/), which are publicly available databases.

## References

[1] Bray F, Ferlay J, Soerjomataram I, Siegel RL, Torre LA and Jemal A (2018) Global cancer statistics 2018: GLOBOCAN estimates of incidence and mortality worldwide for 36 cancers in 185 countries. CA Cancer J Clin 68, 394–424. 30207593 10.3322/caac.21492

[2] Nishihara R, Ogino S and Chan AT (2013) Colorectal‐cancer incidence and mortality after screening. N Engl J Med 369, 2355. 10.1056/NEJMc1313116 24350357

[3] Shaukat A, Mongin SJ, Geisser MS, Lederle FA, Bond JH, Mandel JS and Church TR (2013) Long‐term mortality after screening for colorectal cancer. N Engl J Med 369, 1106–1114. 24047060 10.1056/NEJMoa1300720

[4] Carethers JM and Jung BH (2015) Genetics and genetic biomarkers in sporadic colorectal cancer. Gastroenterology 149, 1177–1190 e1173. 26216840 10.1053/j.gastro.2015.06.047 PMC4589489

[5] Boland CR, Sinicrope FA, Brenner DE and Carethers JM (2000) Colorectal cancer prevention and treatment. Gastroenterology 118, S115–S128. 10868902 10.1016/s0016-5085(00)70010-2

[6] Chang W, Gao X, Han Y, Du Y, Liu Q, Wang L, Tan X, Zhang Q, Liu Y, Zhu Y*et al*. (2014) Gene expression profiling‐derived immunohistochemistry signature with high prognostic value in colorectal carcinoma. Gut 63, 1457–1467. 24173294 10.1136/gutjnl-2013-305475

[7] Haggar FA and Boushey RP (2009) Colorectal cancer epidemiology: incidence, mortality, survival, and risk factors. Clin Colon Rectal Surg 22, 191–197. 21037809 10.1055/s-0029-1242458 PMC2796096

[8] Xiong Y, Wang R, Peng L, You W, Wei J, Zhang S, Wu X, Guo J, Xu J, Lv Z*et al*. (2017) An integrated lncRNA, microRNA and mRNA signature to improve prognosis prediction of colorectal cancer. Oncotarget 8, 85463–85478. 29156733 10.18632/oncotarget.20013 PMC5689623

[9] Zinicola R, Pedrazzi G, Haboubi N and Nicholls RJ (2017) The degree of extramural spread of T3 rectal cancer: a plea to the UICC and AJCC. Colorectal Dis 19, 310. 27418312 10.1111/codi.13456

[10] Sinicrope FA, Okamoto K, Kasi PM and Kawakami H (2016) Molecular biomarkers in the personalized treatment of colorectal cancer. Clin Gastroenterol Hepatol 14, 651–658. 26872400 10.1016/j.cgh.2016.02.008 PMC4836987

[11] Van Schaeybroeck S, Allen WL, Turkington RC and Johnston PG (2011) Implementing prognostic and predictive biomarkers in CRC clinical trials. Nat Rev Clin Oncol 8, 222–232. 21321566 10.1038/nrclinonc.2011.15

[12] Okugawa Y, Grady WM and Goel A (2015) Epigenetic alterations in colorectal cancer: emerging biomarkers. Gastroenterology 149, 1204–1225 e1212. 26216839 10.1053/j.gastro.2015.07.011 PMC4589488

[13] Kristensen VN, Lingjærde OC, Russnes HG, Vollan HK, Frigessi A and Børresen‐Dale AL (2014) Principles and methods of integrative genomic analyses in cancer. Nat Rev Cancer 14, 299–313. 24759209 10.1038/nrc3721

[14] Cancer Genome Atlas Network (2012) Comprehensive molecular characterization of human colon and rectal cancer. Nature 487, 330–337. 22810696 10.1038/nature11252 PMC3401966

[15] Lee MS, Menter DG and Kopetz S (2017) Right versus left colon cancer biology: integrating the consensus molecular subtypes. J Natl Compr Canc Netw 15, 411–419. 28275039 10.6004/jnccn.2017.0038

[16] Ogino S, Cantor M, Kawasaki T, Brahmandam M, Kirkner GJ, Weisenberger DJ, Campan M, Laird PW, Loda M and Fuchs CS (2006) CpG island methylator phenotype (CIMP) of colorectal cancer is best characterised by quantitative DNA methylation analysis and prospective cohort studies. Gut 55, 1000–1006. 16407376 10.1136/gut.2005.082933 PMC1856352

[17] Zhu Y, Qiu P and Ji Y (2014) TCGA‐assembler: open‐source software for retrieving and processing TCGA data. Nat Methods 11, 599–600. 24874569 10.1038/nmeth.2956 PMC4387197

[18] Sanford T, Meng MV, Railkar R, Agarwal PK and Porten SP (2018) Integrative analysis of the epigenetic basis of muscle‐invasive urothelial carcinoma. Clin Epigenetics 10, 19. 29456764 10.1186/s13148-018-0451-x PMC5809922

[19] Robinson MD, McCarthy DJ and Smyth GK (2010) edgeR: a Bioconductor package for differential expression analysis of digital gene expression data. Bioinformatics 26, 139–140. 19910308 10.1093/bioinformatics/btp616 PMC2796818

[20] Kruppa J and Jung K (2017) Automated multigroup outlier identification in molecular high‐throughput data using bagplots and gemplots. BMC Bioinformatics 18, 232. 28464790 10.1186/s12859-017-1645-5 PMC5414140

[21] Sabates‐Bellver J, Van der Flier LG, de Palo M, Cattaneo E, Maake C, Rehrauer H, Laczko E, Kurowski MA, Bujnicki JM, Menigatti M*et al*. (2007) Transcriptome profile of human colorectal adenomas. Mol Cancer Res 5, 1263–1275. 18171984 10.1158/1541-7786.MCR-07-0267

[22] Naumov VA, Generozov EV, Zaharjevskaya NB, Matushkina DS, Larin AK, Chernyshov SV, Alekseev MV, Shelygin YA and Govorun VM (2013) Genome‐scale analysis of DNA methylation in colorectal cancer using Infinium HumanMethylation450 BeadChips. Epigenetics 8, 921–934. 23867710 10.4161/epi.25577 PMC3883769

[23] Gevaert O (2015) MethylMix: an R package for identifying DNA methylation‐driven genes. Bioinformatics 31, 1839–1841. 25609794 10.1093/bioinformatics/btv020 PMC4443673

[24] Gevaert O, Tibshirani R and Plevritis SK (2015) Pancancer analysis of DNA methylation‐driven genes using MethylMix. Genome Biol 16, 17. 25631659 10.1186/s13059-014-0579-8 PMC4365533

[25] Tibshirani R (2011) Regression shrinkage and selection via the lasso: a retrospective. J R Stat Soc Series B Stat Methodol 73, 273–282.

[26] Engebretsen S and Bohlin J (2019) Statistical predictions with glmnet. Clin Epigenetics 11, 123. 31443682 10.1186/s13148-019-0730-1 PMC6708235

[27] Harrell FE Jr, Lee KL and Mark DB (1996) Multivariable prognostic models: issues in developing models, evaluating assumptions and adequacy, and measuring and reducing errors. Stat Med 15, 361–387. 8668867 10.1002/(SICI)1097-0258(19960229)15:4<361::AID-SIM168>3.0.CO;2-4

[28] Pan Y, Song Y, Cheng L, Xu H and Liu J (2019) Analysis of methylation‐driven genes for predicting the prognosis of patients with head and neck squamous cell carcinoma. J Cell Biochem 120, 19482–19495. 31264288 10.1002/jcb.29252

[29] Subramanian A, Tamayo P, Mootha VK, Mukherjee S, Ebert BL, Gillette MA, Paulovich A, Pomeroy SL, Golub TR, Lander ES*et al*. (2005) Gene set enrichment analysis: a knowledge‐based approach for interpreting genome‐wide expression profiles. Proc Natl Acad Sci USA 102, 15545–15550. 16199517 10.1073/pnas.0506580102 PMC1239896

[30] Guo Y, Peng Y, Gao D, Zhang M, Yang W, Linghu E, Herman JG, Fuks F, Dong G and Guo M (2017) Silencing HOXD10 by promoter region hypermethylation activates ERK signaling in hepatocellular carcinoma. Clin Epigenetics 9, 116. 29075359 10.1186/s13148-017-0412-9 PMC5654145

[31] Herman JG, Graff JR, Myohanen S, Nelkin BD and Baylin SB (1996) Methylation‐specific PCR: a novel PCR assay for methylation status of CpG islands. Proc Natl Acad Sci USA 93, 9821–9826. 8790415 10.1073/pnas.93.18.9821 PMC38513

[32] Dienstmann R, Mason MJ, Sinicrope FA, Phipps AI, Tejpar S, Nesbakken A, Danielsen SA, Sveen A, Buchanan DD, Clendenning M*et al*. (2017) Prediction of overall survival in stage II and III colon cancer beyond TNM system: a retrospective, pooled biomarker study. Ann Oncol 28, 1023–1031. 28453697 10.1093/annonc/mdx052 PMC5406760

[33] Saharinen P, Eklund L, Pulkki K, Bono P and Alitalo K (2011) VEGF and angiopoietin signaling in tumor angiogenesis and metastasis. Trends Mol Med 17, 347–362. 21481637 10.1016/j.molmed.2011.01.015

[34] Piechutta M and Berghoff AS (2019) New emerging targets in cancer immunotherapy: the role of cluster of differentiation 40 (CD40/TNFR5). ESMO Open 4, e000510. 31275618 10.1136/esmoopen-2019-000510 PMC6579575

[35] Jones PA and Baylin SB (2007) The epigenomics of cancer. Cell 128, 683–692. 17320506 10.1016/j.cell.2007.01.029 PMC3894624

[36] Weisenberger DJ, Siegmund KD, Campan M, Young J, Long TI, Faasse MA, Kang GH, Widschwendter M, Weener D, Buchanan D*et al*. (2006) CpG island methylator phenotype underlies sporadic microsatellite instability and is tightly associated with BRAF mutation in colorectal cancer. Nat Genet 38, 787–793. 16804544 10.1038/ng1834

[37] Coppede F, Lopomo A, Spisni R and Migliore L (2014) Genetic and epigenetic biomarkers for diagnosis, prognosis and treatment of colorectal cancer. World J Gastroenterol 20, 943–956. 24574767 10.3748/wjg.v20.i4.943 PMC3921546

[38] Sakai E, Nakajima A and Kaneda A (2014) Accumulation of aberrant DNA methylation during colorectal cancer development. World J Gastroenterol 20, 978–987. 24574770 10.3748/wjg.v20.i4.978 PMC3921549

[39] Juo YY, Johnston FM, Zhang DY, Juo HH, Wang H, Pappou EP, Yu T, Easwaran H, Baylin S, van Engeland M*et al*. (2014) Prognostic value of CpG island methylator phenotype among colorectal cancer patients: a systematic review and meta‐analysis. Ann Oncol 25, 2314–2327. 24718889 10.1093/annonc/mdu149 PMC4239805

[40] Inamura K, Togashi Y, Ninomiya H, Shimoji T, Noda T and Ishikawa Y (2008) HOXB2, an adverse prognostic indicator for stage I lung adenocarcinomas, promotes invasion by transcriptional regulation of metastasis‐related genes in HOP‐62 non‐small cell lung cancer cells. Anticancer Res 28, 2121–2127. 18751384

[41] Inamura K, Togashi Y, Okui M, Ninomiya H, Hiramatsu M, Satoh Y, Okumura S, Nakagawa K, Shimoji T, Noda T*et al*. (2007) HOXB2 as a novel prognostic indicator for stage I lung adenocarcinomas. J Thorac Oncol 2, 802–807. 17805056 10.1097/JTO.0b013e3181461987

[42] Marsit CJ, Houseman EA, Christensen BC, Gagne L, Wrensch MR, Nelson HH, Wiemels J, Zheng S, Wiencke JK, Andrew AS*et al*. (2010) Identification of methylated genes associated with aggressive bladder cancer. PLoS One 5, e12334. 20808801 10.1371/journal.pone.0012334 PMC2925945

[43] Li X, Wang J, Zhang X, Zeng Y, Liang L and Ding Y (2012) Overexpression of RhoGDI2 correlates with tumor progression and poor prognosis in colorectal carcinoma. Ann Surg Oncol 19, 145–153. 21861235 10.1245/s10434-011-1944-4

[44] Tapper J, Kettunen E, El‐Rifai W, Seppala M, Andersson LC and Knuutila S (2001) Changes in gene expression during progression of ovarian carcinoma. Cancer Genet Cytogenet 128, 1–6. 11454421 10.1016/s0165-4608(01)00386-7

[45] Zhang Y and Zhang B (2006) D4‐GDI, a Rho GTPase regulator, promotes breast cancer cell invasiveness. Cancer Res 66, 5592–5598. 16740694 10.1158/0008-5472.CAN-05-4004

[46] Cho HJ, Baek KE, Park SM, Kim IK, Choi YL, Cho HJ, Nam IK, Hwang EM, Park JY, Han JY*et al*. (2009) RhoGDI2 expression is associated with tumor growth and malignant progression of gastric cancer. Clin Cancer Res 15, 2612–2619. 19351766 10.1158/1078-0432.CCR-08-2192

[47] Ma L, Xu G, Sotnikova A, Szczepanowski M, Giefing M, Krause K, Krams M, Siebert R, Jin J and Klapper W (2007) Loss of expression of LyGDI (ARHGDIB), a rho GDP‐dissociation inhibitor, in Hodgkin lymphoma. Br J Haematol 139, 217–223. 17897297 10.1111/j.1365-2141.2007.06782.x

[48] Seraj MJ, Harding MA, Gildea JJ, Welch DR and Theodorescu D (2000) The relationship of BRMS1 and RhoGDI2 gene expression to metastatic potential in lineage related human bladder cancer cell lines. Clin Exp Metastasis 18, 519–525. 11592309 10.1023/a:1011819621859

[49] Theodorescu D, Sapinoso LM, Conaway MR, Oxford G, Hampton GM and Frierson HF Jr (2004) Reduced expression of metastasis suppressor RhoGDI2 is associated with decreased survival for patients with bladder cancer. Clin Cancer Res 10, 3800–3806. 15173088 10.1158/1078-0432.CCR-03-0653

[50] Said N, Sanchez‐Carbayo M, Smith SC and Theodorescu D (2012) RhoGDI2 suppresses lung metastasis in mice by reducing tumor versican expression and macrophage infiltration. J Clin Invest 122, 1503–1518. 22406535 10.1172/JCI61392 PMC3314474

[51] Ge YX, Wang CH, Hu FY, Pan LX, Min J, Niu KY, Zhang L, Li J and Xu T (2018) New advances of TMEM88 in cancer initiation and progression, with special emphasis on Wnt signaling pathway. J Cell Physiol 233, 79–87. 28181235 10.1002/jcp.25853

[52] de Leon M, Cardenas H, Vieth E, Emerson R, Segar M, Liu Y, Nephew K and Matei D (2016) Transmembrane protein 88 (TMEM88) promoter hypomethylation is associated with platinum resistance in ovarian cancer. Gynecol Oncol 142, 539–547. 27374141 10.1016/j.ygyno.2016.06.017 PMC4993677

[53] Ayala I, Giacchetti G, Caldieri G, Attanasio F, Mariggio S, Tete S, Polishchuk R, Castronovo V and Buccione R (2009) Faciogenital dysplasia protein Fgd1 regulates invadopodia biogenesis and extracellular matrix degradation and is up‐regulated in prostate and breast cancer. Cancer Res 69, 747–752. 19141649 10.1158/0008-5472.CAN-08-1980

[54] Das A, Dickinson DJ, Wood CC, Goldstein B and Slep KC (2015) Crescerin uses a TOG domain array to regulate microtubules in the primary cilium. Mol Biol Cell 26, 4248–4264. 26378256 10.1091/mbc.E15-08-0603 PMC4642858

[55] van Kooten C and Banchereau J (2000) CD40‐CD40 ligand. J Leukoc Biol 67, 2–17. 10647992 10.1002/jlb.67.1.2

[56] Tewari R, Choudhury SR, Mehta VS and Sen E (2012) TNFalpha regulates the localization of CD40 in lipid rafts of glioma cells. Mol Biol Rep 39, 8695–8699. 22699883 10.1007/s11033-012-1726-5

[57] Xie F, Shi Q, Wang Q, Ge Y, Chen Y, Zuo J, Gu Y, Deng H, Mao H, Hu Z*et al*. (2010) CD40 is a regulator for vascular endothelial growth factor in the tumor microenvironment of glioma. J Neuroimmunol 222, 62–69. 20303602 10.1016/j.jneuroim.2009.12.004

[58] Zhou Y, Zhou SX, Gao L and Li XA (2016) Regulation of CD40 signaling in colon cancer cells and its implications in clinical tissues. Cancer Immunol Immunother 65, 919–929. 27262846 10.1007/s00262-016-1847-0 PMC11028916

[59] Yamaguchi H, Tanaka F, Sadanaga N, Ohta M, Inoue H and Mori M (2003) Stimulation of CD40 inhibits Fas‐ or chemotherapy‐mediated apoptosis and increases cell motility in human gastric carcinoma cells. Int J Oncol 23, 1697–1702. 14612943

[60] Vonderheide RH (2018) The immune revolution: a case for priming, not checkpoint. Cancer Cell 33, 563–569. 29634944 10.1016/j.ccell.2018.03.008 PMC5898647

[61] Ma R, Feng N, Yu X, Lin H, Zhang X, Shi O, Zhang H, Zhang S, Li L, Zheng M*et al*. (2017) Promoter methylation of Wnt/beta‐Catenin signal inhibitor TMEM88 is associated with unfavorable prognosis of non‐small cell lung cancer. Cancer Biol Med 14, 377–386. 29372104 10.20892/j.issn.2095-3941.2017.0061 PMC5765437

[62] Nagata H, Kozaki KI, Muramatsu T, Hiramoto H, Tanimoto K, Fujiwara N, Imoto S, Ichikawa D, Otsuji E, Miyano S*et al*. (2017) Genome‐wide screening of DNA methylation associated with lymph node metastasis in esophageal squamous cell carcinoma. Oncotarget 8, 37740–37750. 28465481 10.18632/oncotarget.17147 PMC5514945

[63] Xavier FC, Destro MF, Duarte CM and Nunes FD (2014) Epigenetic repression of HOXB cluster in oral cancer cell lines. Arch Oral Biol 59, 783–789. 24859765 10.1016/j.archoralbio.2014.05.001

[64] Cai C, Xie X, Zhou J, Fang X, Wang F and Wang M (2020) Identification of TAF1, SAT1, and ARHGEF9 as DNA methylation biomarkers for hepatocellular carcinoma. J Cell Physiol 235, 611–618. 31283007 10.1002/jcp.28999

[65] Wang L, Shi J, Huang Y, Liu S, Zhang J, Ding H, Yang J and Chen Z (2019) A six‐gene prognostic model predicts overall survival in bladder cancer patients. Cancer Cell Int 19, 229. 31516386 10.1186/s12935-019-0950-7 PMC6729005

[66] Huang T, Yang J and Cai YD (2015) Novel candidate key drivers in the integrative network of genes, microRNAs, methylations, and copy number variations in squamous cell lung carcinoma. Biomed Res Int 2015, 358125. 25802847 10.1155/2015/358125 PMC4352729

[67] Zeller C, Dai W, Steele NL, Siddiq A, Walley AJ, Wilhelm‐Benartzi CS, Rizzo S, van der Zee A, Plumb JA and Brown R (2012) Candidate DNA methylation drivers of acquired cisplatin resistance in ovarian cancer identified by methylome and expression profiling. Oncogene 31, 4567–4576. 22249249 10.1038/onc.2011.611

