# Supplementary figures and images for: A methylation‐driven gene panel predicts survival in patients with colon cancer
Source: FEBS Open Bio. 2021 Jul 28;11(9):2490–506. doi: 10.1002/2211-5463.13242 (PMC8409306; doi:10.1002/2211-5463.13242)

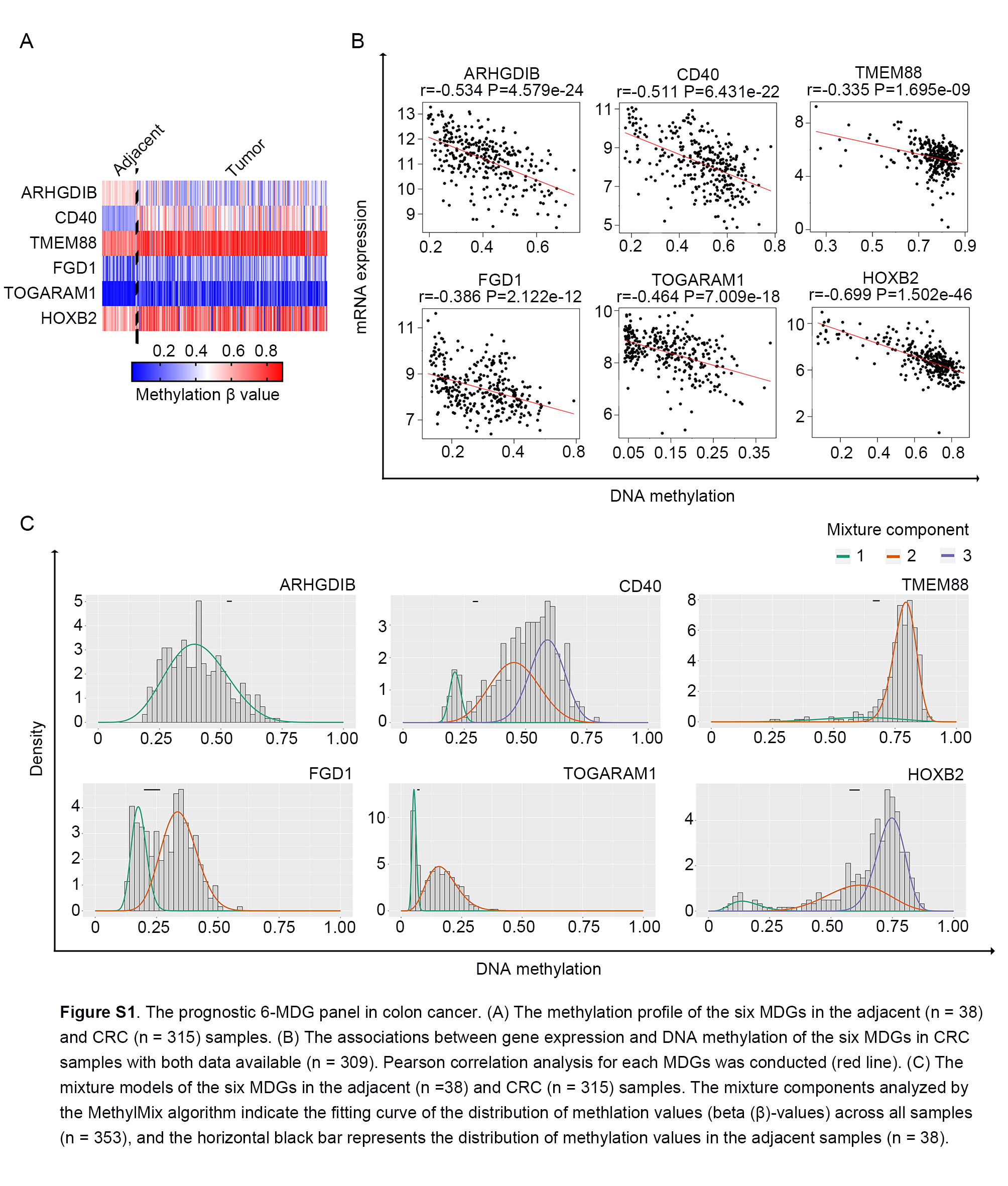

Supplement: Supplementary file 1 — Fig. S1. The prognostic 6‐MDG panel in colon cancer. (A) The methylation profile of the six MDGs in the adjacent (n = 38) and CRC (n = 315) samples. (B) The associations between gene expression and DNA methylation of the six MDGs in CRC samples with both data available (n = 309). Pearson correlation analysis for each MDGs was conducted (red line). (C) The mixture models of the six MDGs in the adjacent (n = 38) and CRC (n = 315) samples. The mixture components analyzed by the MethylMix algorithm indicate the fitting curve of the distribution of methylation values (beta (β)‐values) across all samples (n = 353), and the horizontal black bar represents the distribution of methylation values in the adjacent samples (n = 38). [file FEB4-11-2490-s002.jpg]
