# Supplementary material for: A methylation‐driven gene panel predicts survival in patients with colon cancer
Source: FEBS Open Bio. 2021 Jul 28;11(9):2490–506. doi: 10.1002/2211-5463.13242 (PMC8409306; doi:10.1002/2211-5463.13242)
Supplement: Supplementary file 2 — Table S1. Primers used in this study. [file FEB4-11-2490-s005.docx]

**Table S1.** Primers used in this study.

| Primers | Sequence (5’-3’) |
| --- | --- |
| rtPCR-CD40 (F) | GATACCATCTGCGAGCCCTG |
| rtPCR-CD40 (R) | GCTTCTTGGCCACCTTTTTG |
| rtPCR-GAPDH (F) | CAAGGTCATCCATGACAACTTTG |
| rtPCR-GAPDH (R) | GTCCACCACCCTGTTGCTGTAG |
| MSP-CD40 (MF) | TTTCGATAGGTGGATCGC |
| MSP-CD40 (MR) | CGACAAAACCACTAAACGC |
| MSP-CD40 (UF) | TTTTTTTGATAGGTGGATTGT |
| MSP-CD40 (UR) | CAACAAAACCACTAAACACCCA |
| BSSQ-CD40 (F) | AATGTTTTGGGGAAATTTTTG |
| BSSQ-CD40 (R) | AATCAACCAAAAAAACCTCTTC |
