# Supplementary material for: A methylation‐driven gene panel predicts survival in patients with colon cancer
Source: FEBS Open Bio. 2021 Jul 28;11(9):2490–506. doi: 10.1002/2211-5463.13242 (PMC8409306; doi:10.1002/2211-5463.13242)
Supplement: Supplementary file 3 — Table S2. Methylation‐driven genes identified in colon cancer patients. [file FEB4-11-2490-s004.docx]

**Table S2.** Methylation-driven genes identified in colon cancer patients.

| Gene | Mean Adjacent  (β value) | Mean Tumor  (β value) | Log  fold change | Adjusted  P value | Pearson r | P value |
| --- | --- | --- | --- | --- | --- | --- |
| ADHFE1 | 0.22211 | 0.333409 | 0.586015 | 1.41E-20 | -0.42399 | 7.20E-15 |
| CNNM1 | 0.448226 | 0.599136 | 0.418655 | 1.83E-20 | -0.39826 | 3.77E-13 |
| CCDC8 | 0.375762 | 0.561206 | 0.578711 | 2.33E-20 | -0.40642 | 1.11E-13 |
| FAM43B | 0.257479 | 0.485639 | 0.91543 | 2.86E-20 | -0.37457 | 1.08E-11 |
| CHST2 | 0.310506 | 0.569165 | 0.874224 | 3.45E-20 | -0.35855 | 8.94E-11 |
| MSC | 0.225841 | 0.533391 | 1.239883 | 4.46E-20 | -0.48754 | 8.54E-20 |
| GDF6 | 0.27584 | 0.504509 | 0.871046 | 5.65E-20 | -0.32256 | 6.90E-09 |
| MAFB | 0.125342 | 0.299448 | 1.256432 | 6.05E-20 | -0.3389 | 1.03E-09 |
| ADAMTS5 | 0.359317 | 0.550496 | 0.615476 | 8.21E-20 | -0.38045 | 4.80E-12 |
| ZNF625 | 0.206618 | 0.518751 | 1.328075 | 8.78E-20 | -0.50963 | 9.23E-22 |
| CNRIP1 | 0.326215 | 0.563911 | 0.789647 | 9.40E-20 | -0.54306 | 5.03E-25 |
| DOK5 | 0.129256 | 0.353764 | 1.452553 | 1.08E-19 | -0.3585 | 9.00E-11 |
| RP11-710C12.1 | 0.118098 | 0.445868 | 1.916637 | 1.08E-19 | -0.43626 | 9.65E-16 |
| HAND2 | 0.215082 | 0.456622 | 1.086114 | 1.19E-19 | -0.35025 | 2.56E-10 |
| CDO1 | 0.326709 | 0.500998 | 0.616799 | 1.48E-19 | -0.43593 | 1.02E-15 |
| RARRES2 | 0.481251 | 0.647063 | 0.427115 | 2.01E-19 | -0.42847 | 3.49E-15 |
| PCDH8 | 0.269572 | 0.527044 | 0.967251 | 2.14E-19 | -0.53638 | 2.42E-24 |
| DPY19L2 | 0.125869 | 0.501559 | 1.994498 | 2.22E-19 | -0.32564 | 4.86E-09 |
| ZNF568 | 0.102324 | 0.371896 | 1.86175 | 2.54E-19 | -0.602 | 9.39E-32 |
| SLIT2 | 0.294605 | 0.433616 | 0.557639 | 3.00E-19 | -0.39544 | 5.69E-13 |
| HLX | 0.278108 | 0.460831 | 0.728589 | 3.10E-19 | -0.3084 | 3.28E-08 |
| RP11-573G6.4 | 0.152515 | 0.424481 | 1.47675 | 4.18E-19 | -0.40429 | 1.54E-13 |
| ZNF135 | 0.541545 | 0.671442 | 0.310182 | 4.70E-19 | -0.46716 | 4.21E-18 |
| KCNA3 | 0.425529 | 0.658839 | 0.630668 | 4.94E-19 | -0.45543 | 3.54E-17 |
| BNIP3 | 0.492573 | 0.601488 | 0.288198 | 4.98E-19 | -0.42957 | 2.92E-15 |
| AVPR1A | 0.236577 | 0.414885 | 0.810401 | 5.93E-19 | -0.42524 | 5.89E-15 |
| THBD | 0.179207 | 0.458063 | 1.353917 | 5.93E-19 | -0.34642 | 4.12E-10 |
| EFS | 0.287074 | 0.525249 | 0.871579 | 6.66E-19 | -0.31026 | 2.69E-08 |
| SOX17 | 0.335641 | 0.604658 | 0.849201 | 7.23E-19 | -0.53868 | 1.41E-24 |
| FBLIM1 | 0.260323 | 0.452222 | 0.796728 | 7.86E-19 | -0.33204 | 2.32E-09 |
| TWIST1 | 0.264685 | 0.511306 | 0.949907 | 8.05E-19 | -0.41759 | 1.99E-14 |
| CLIP4 | 0.345491 | 0.468529 | 0.439493 | 8.12E-19 | -0.38962 | 1.32E-12 |
| ST6GALNAC5 | 0.294658 | 0.431103 | 0.548994 | 8.97E-19 | -0.39568 | 5.50E-13 |
| CHL1 | 0.435347 | 0.586013 | 0.428768 | 9.12E-19 | -0.32746 | 3.94E-09 |
| RP11-23P13.6 | 0.404315 | 0.278264 | -0.53903 | 1.21E-18 | -0.62078 | 3.32E-34 |
| ZNF331 | 0.348084 | 0.489257 | 0.491157 | 1.23E-18 | -0.65832 | 1.23E-39 |
| AQP1 | 0.340448 | 0.491653 | 0.530207 | 1.40E-18 | -0.41514 | 2.92E-14 |
| ZNF134 | 0.231635 | 0.429411 | 0.890508 | 1.97E-18 | -0.81888 | 9.23E-76 |
| LAYN | 0.248953 | 0.395366 | 0.667316 | 2.11E-18 | -0.39779 | 4.04E-13 |
| PCDH10 | 0.250119 | 0.518707 | 1.052308 | 2.20E-18 | -0.41169 | 4.98E-14 |
| EVC | 0.20941 | 0.429812 | 1.037377 | 2.52E-18 | -0.3099 | 2.79E-08 |
| AC021218.2 | 0.620755 | 0.431067 | -0.52611 | 2.54E-18 | -0.46167 | 1.15E-17 |
| SPG20 | 0.287238 | 0.432621 | 0.59086 | 3.07E-18 | -0.46331 | 8.56E-18 |
| LRFN5 | 0.177542 | 0.363256 | 1.032821 | 4.01E-18 | -0.41513 | 2.92E-14 |
| GALNT13 | 0.24803 | 0.433766 | 0.806403 | 4.32E-18 | -0.50263 | 4.02E-21 |
| TLL1 | 0.187427 | 0.408324 | 1.123389 | 4.53E-18 | -0.32534 | 5.03E-09 |
| HCG4 | 0.16635 | 0.283928 | 0.771301 | 5.08E-18 | -0.39508 | 6.00E-13 |
| LINC00944 | 0.301977 | 0.18319 | -0.7211 | 5.50E-18 | -0.45883 | 1.93E-17 |
| TGFBI | 0.393516 | 0.290328 | -0.43874 | 6.02E-18 | -0.63099 | 1.32E-35 |
| ELAVL2 | 0.209332 | 0.400964 | 0.937682 | 6.07E-18 | -0.63507 | 3.49E-36 |
| ZNF813 | 0.13843 | 0.278735 | 1.009735 | 6.47E-18 | -0.76234 | 8.90E-60 |
| SUSD5 | 0.45245 | 0.610537 | 0.43232 | 8.65E-18 | -0.37952 | 5.46E-12 |
| PPP1R16B | 0.377447 | 0.520115 | 0.462558 | 1.14E-17 | -0.37307 | 1.32E-11 |
| PROM2 | 0.613156 | 0.715257 | 0.222208 | 1.21E-17 | -0.4554 | 3.56E-17 |
| TM6SF1 | 0.241736 | 0.488834 | 1.015911 | 1.26E-17 | -0.34676 | 3.95E-10 |
| PTF1A | 0.192344 | 0.456105 | 1.245678 | 1.49E-17 | -0.47759 | 5.92E-19 |
| B3GNT7 | 0.33628 | 0.444632 | 0.402948 | 1.78E-17 | -0.45624 | 3.06E-17 |
| CCNA1 | 0.307389 | 0.487843 | 0.66635 | 2.26E-17 | -0.34468 | 5.10E-10 |
| PTGFR | 0.360189 | 0.499167 | 0.47077 | 2.53E-17 | -0.34773 | 3.50E-10 |
| CCDC169 | 0.132721 | 0.396024 | 1.577187 | 2.87E-17 | -0.48681 | 9.87E-20 |
| DMRTA1 | 0.111718 | 0.385179 | 1.78567 | 2.96E-17 | -0.41192 | 4.81E-14 |
| TRBC2 | 0.216871 | 0.324088 | 0.579547 | 3.21E-17 | -0.34652 | 4.07E-10 |
| NEFH | 0.377837 | 0.560164 | 0.568085 | 4.23E-17 | -0.40915 | 7.35E-14 |
| GBGT1 | 0.363887 | 0.469565 | 0.367834 | 4.27E-17 | -0.62965 | 2.02E-35 |
| ZNF582 | 0.187968 | 0.457055 | 1.281878 | 4.47E-17 | -0.64666 | 7.24E-38 |
| ZSCAN23 | 0.159966 | 0.37671 | 1.23569 | 4.54E-17 | -0.59732 | 3.62E-31 |
| CAHM | 0.062739 | 0.277352 | 2.144271 | 4.73E-17 | -0.41471 | 3.12E-14 |
| ZNF471 | 0.175619 | 0.471428 | 1.424589 | 5.41E-17 | -0.70033 | 1.02E-46 |
| TDRP | 0.361444 | 0.49267 | 0.446848 | 5.58E-17 | -0.5301 | 1.02E-23 |
| USP54 | 0.529932 | 0.643502 | 0.280135 | 5.94E-17 | -0.36647 | 3.19E-11 |
| ZNF334 | 0.233865 | 0.473482 | 1.017631 | 6.04E-17 | -0.58329 | 1.81E-29 |
| ZNF569 | 0.12698 | 0.316155 | 1.316031 | 9.15E-17 | -0.66467 | 1.24E-40 |
| ADCYAP1 | 0.282807 | 0.4812 | 0.766817 | 1.07E-16 | -0.42227 | 9.48E-15 |
| AC009014.3 | 0.182035 | 0.285005 | 0.646766 | 1.95E-16 | -0.54119 | 7.83E-25 |
| ZNF454 | 0.336029 | 0.511319 | 0.605639 | 2.94E-16 | -0.4625 | 9.92E-18 |
| ZNF726 | 0.212368 | 0.521906 | 1.297224 | 3.57E-16 | -0.45514 | 3.73E-17 |
| C17orf107 | 0.241084 | 0.440785 | 0.870539 | 4.22E-16 | -0.34616 | 4.25E-10 |
| CTD-2368P22.1 | 0.135784 | 0.283184 | 1.060423 | 4.39E-16 | -0.59958 | 1.89E-31 |
| ZNF793 | 0.198227 | 0.493093 | 1.314708 | 4.56E-16 | -0.72478 | 1.94E-51 |
| FAM218A | 0.263632 | 0.508502 | 0.947726 | 4.85E-16 | -0.52399 | 4.05E-23 |
| FOXG1 | 0.230517 | 0.500317 | 1.11797 | 5.12E-16 | -0.53188 | 6.82E-24 |
| ZNF347 | 0.207705 | 0.377206 | 0.860816 | 5.24E-16 | -0.64739 | 5.64E-38 |
| AKR1B1 | 0.252824 | 0.515662 | 1.028291 | 9.36E-16 | -0.6359 | 2.66E-36 |
| SMO | 0.175847 | 0.303663 | 0.788149 | 1.16E-15 | -0.53173 | 7.06E-24 |
| SLFN13 | 0.311359 | 0.417692 | 0.423859 | 1.54E-15 | -0.5938 | 9.83E-31 |
| AZGP1 | 0.636179 | 0.496236 | -0.35841 | 1.91E-15 | -0.38399 | 2.93E-12 |
| ZNF677 | 0.336595 | 0.471012 | 0.48475 | 2.15E-15 | -0.53624 | 2.50E-24 |
| ZNF43 | 0.047702 | 0.28223 | 2.564746 | 2.27E-15 | -0.80198 | 1.91E-70 |
| PHOX2B | 0.308825 | 0.444065 | 0.523981 | 2.41E-15 | -0.38813 | 1.63E-12 |
| RP11-430H10.1 | 0.291244 | 0.472612 | 0.698426 | 2.48E-15 | -0.50222 | 4.38E-21 |
| RNF165 | 0.247706 | 0.398971 | 0.687653 | 2.78E-15 | -0.3963 | 5.02E-13 |
| LDOC1 | 0.322599 | 0.566329 | 0.8119 | 3.09E-15 | -0.50414 | 2.93E-21 |
| PABPC5 | 0.570445 | 0.744344 | 0.383882 | 3.62E-15 | -0.46449 | 6.89E-18 |
| CTB-41I6.1 | 0.369599 | 0.543247 | 0.55565 | 3.67E-15 | -0.34585 | 4.42E-10 |
| AP000251.3 | 0.059829 | 0.256285 | 2.098828 | 3.75E-15 | -0.64853 | 3.81E-38 |
| SOX1 | 0.225423 | 0.418104 | 0.89123 | 4.70E-15 | -0.31645 | 1.36E-08 |
| CMTM3 | 0.135102 | 0.370138 | 1.454019 | 5.87E-15 | -0.46571 | 5.51E-18 |
| WDR17 | 0.154275 | 0.338883 | 1.135284 | 6.14E-15 | -0.50929 | 9.91E-22 |
| ZNF492 | 0.152213 | 0.316868 | 1.057788 | 6.42E-15 | -0.52997 | 1.05E-23 |
| CLIC6 | 0.335631 | 0.437172 | 0.381327 | 7.61E-15 | -0.68929 | 9.71E-45 |
| ZNF85 | 0.211648 | 0.449751 | 1.087458 | 8.77E-15 | -0.69136 | 4.21E-45 |
| TBX18 | 0.21248 | 0.478281 | 1.170532 | 1.08E-14 | -0.77036 | 9.20E-62 |
| CTC-444N24.13 | 0.134137 | 0.412012 | 1.618982 | 1.15E-14 | -0.53063 | 9.06E-24 |
| AC009237.8 | 0.528201 | 0.628278 | 0.250317 | 1.33E-14 | -0.69691 | 4.30E-46 |
| CD34 | 0.499743 | 0.615546 | 0.300681 | 2.12E-14 | -0.43706 | 8.45E-16 |
| FAS | 0.563243 | 0.726482 | 0.367168 | 2.72E-14 | -0.3866 | 2.03E-12 |
| TIGD7 | 0.411405 | 0.568526 | 0.466667 | 3.33E-14 | -0.46513 | 6.12E-18 |
| ZNF730 | 0.111033 | 0.313342 | 1.496752 | 3.90E-14 | -0.63504 | 3.53E-36 |
| FOXD2 | 0.147411 | 0.268712 | 0.866221 | 4.08E-14 | -0.54696 | 1.98E-25 |
| NFE2L3 | 0.228377 | 0.184563 | -0.30731 | 4.23E-14 | -0.5076 | 1.42E-21 |
| ZNF528 | 0.171786 | 0.399444 | 1.217378 | 4.29E-14 | -0.73302 | 3.80E-53 |
| CTD-2666L21.1 | 0.2951 | 0.529818 | 0.844293 | 4.71E-14 | -0.47242 | 1.58E-18 |
| AF186192.1 | 0.206718 | 0.47112 | 1.188428 | 4.92E-14 | -0.5544 | 3.23E-26 |
| EPHX3 | 0.216806 | 0.397142 | 0.87325 | 5.45E-14 | -0.43501 | 1.19E-15 |
| GREB1L | 0.151237 | 0.436817 | 1.530216 | 6.47E-14 | -0.49291 | 2.93E-20 |
| TFAP2C | 0.16014 | 0.253326 | 0.661661 | 6.81E-14 | -0.58183 | 2.69E-29 |
| FADS1 | 0.152174 | 0.274486 | 0.851006 | 8.03E-14 | -0.39144 | 1.02E-12 |
| ZNF880 | 0.187795 | 0.405031 | 1.108874 | 8.51E-14 | -0.66241 | 2.83E-40 |
| HOXA-AS3 | 0.24312 | 0.459927 | 0.919738 | 9.82E-14 | -0.32978 | 3.01E-09 |
| TBX20 | 0.288591 | 0.471448 | 0.708073 | 1.15E-13 | -0.46149 | 1.19E-17 |
| SOX14 | 0.15389 | 0.303404 | 0.979339 | 1.29E-13 | -0.32775 | 3.81E-09 |
| CLDN10 | 0.423706 | 0.528853 | 0.319803 | 1.44E-13 | -0.49775 | 1.10E-20 |
| CIDEB | 0.418134 | 0.544619 | 0.381284 | 1.62E-13 | -0.47899 | 4.53E-19 |
| ANO5 | 0.230687 | 0.472756 | 1.03516 | 1.69E-13 | -0.48627 | 1.10E-19 |
| ZFP82 | 0.238858 | 0.400851 | 0.746915 | 3.67E-13 | -0.73434 | 2.00E-53 |
| AF186192.5 | 0.334931 | 0.445433 | 0.411346 | 5.13E-13 | -0.54823 | 1.46E-25 |
| RP11-394O4.5 | 0.801137 | 0.874209 | 0.125929 | 5.58E-13 | -0.31564 | 1.49E-08 |
| ZNF418 | 0.427209 | 0.577317 | 0.434423 | 5.74E-13 | -0.43309 | 1.64E-15 |
| ZNF788 | 0.223568 | 0.468334 | 1.066827 | 6.33E-13 | -0.57592 | 1.31E-28 |
| FAM200A | 0.043422 | 0.147948 | 1.768593 | 1.20E-12 | -0.4131 | 4.01E-14 |
| TMEM88 | 0.673328 | 0.765796 | 0.185651 | 1.29E-12 | -0.3347 | 1.69E-09 |
| LINC00460 | 0.755409 | 0.619635 | -0.28584 | 2.56E-12 | -0.65769 | 1.54E-39 |
| CD40 | 0.295698 | 0.492809 | 0.736906 | 2.80E-12 | -0.51132 | 6.43E-22 |
| RP11-535M15.1 | 0.44481 | 0.552074 | 0.311672 | 3.00E-12 | -0.37941 | 5.54E-12 |
| STK33 | 0.150067 | 0.27502 | 0.873933 | 3.14E-12 | -0.56414 | 2.81E-27 |
| TCTEX1D1 | 0.343762 | 0.497802 | 0.534163 | 3.16E-12 | -0.32974 | 3.03E-09 |
| SULT4A1 | 0.339544 | 0.456398 | 0.426695 | 3.21E-12 | -0.55322 | 4.32E-26 |
| LINC00346 | 0.379768 | 0.261003 | -0.54105 | 3.27E-12 | -0.51583 | 2.44E-22 |
| ST8SIA4 | 0.230816 | 0.380901 | 0.722669 | 3.30E-12 | -0.31235 | 2.14E-08 |
| ZNF790 | 0.137826 | 0.335336 | 1.28276 | 3.80E-12 | -0.4765 | 7.30E-19 |
| RHOBTB3 | 0.638396 | 0.78796 | 0.303672 | 3.88E-12 | -0.48179 | 2.64E-19 |
| KDR | 0.372811 | 0.507145 | 0.443955 | 4.07E-12 | -0.33729 | 1.25E-09 |
| ZNF256 | 0.222832 | 0.351545 | 0.657753 | 4.78E-12 | -0.75539 | 4.03E-58 |
| C8orf48 | 0.238748 | 0.349352 | 0.549191 | 6.01E-12 | -0.35019 | 2.58E-10 |
| ZNF257 | 0.297608 | 0.436144 | 0.551391 | 7.61E-12 | -0.63256 | 7.90E-36 |
| SNCA | 0.189881 | 0.394809 | 1.056056 | 8.81E-12 | -0.41634 | 2.42E-14 |
| ZNF530 | 0.299052 | 0.440643 | 0.559216 | 1.03E-11 | -0.71562 | 1.30E-49 |
| GPX7 | 0.300164 | 0.419404 | 0.482587 | 1.15E-11 | -0.5993 | 2.05E-31 |
| ZHX1-C8orf76 | 0.621552 | 0.381845 | -0.70289 | 1.40E-11 | -0.30686 | 3.87E-08 |
| HSD17B14 | 0.460166 | 0.586048 | 0.348864 | 1.58E-11 | -0.38208 | 3.83E-12 |
| RERG | 0.241073 | 0.415483 | 0.785322 | 1.60E-11 | -0.36111 | 6.42E-11 |
| AQP5 | 0.371089 | 0.566582 | 0.610518 | 1.74E-11 | -0.73559 | 1.08E-53 |
| ZNF354C | 0.263464 | 0.425177 | 0.69046 | 1.93E-11 | -0.64205 | 3.45E-37 |
| FAM150A | 0.247164 | 0.404499 | 0.710669 | 1.95E-11 | -0.43082 | 2.38E-15 |
| CTD-2554C21.2 | 0.328524 | 0.540396 | 0.71802 | 2.03E-11 | -0.63331 | 6.19E-36 |
| IL2RG | 0.350818 | 0.247412 | -0.5038 | 2.17E-11 | -0.5154 | 2.67E-22 |
| LINC00665 | 0.237906 | 0.404994 | 0.767508 | 3.89E-11 | -0.63716 | 1.76E-36 |
| VSIG2 | 0.473083 | 0.602622 | 0.349161 | 4.18E-11 | -0.68248 | 1.46E-43 |
| TMEM35 | 0.481984 | 0.592806 | 0.298575 | 4.23E-11 | -0.55592 | 2.22E-26 |
| DMRT2 | 0.320851 | 0.432239 | 0.429925 | 4.70E-11 | -0.30436 | 5.05E-08 |
| ZSCAN18 | 0.382142 | 0.496131 | 0.376613 | 7.30E-11 | -0.72548 | 1.40E-51 |
| KLF2 | 0.345364 | 0.454492 | 0.396137 | 8.68E-11 | -0.51247 | 5.03E-22 |
| AC016747.3 | 0.412853 | 0.536712 | 0.378519 | 1.09E-10 | -0.61563 | 1.62E-33 |
| HCG4P5 | 0.076279 | 0.100745 | 0.401347 | 1.16E-10 | -0.33734 | 1.24E-09 |
| CLEC14A | 0.448033 | 0.55113 | 0.29879 | 1.27E-10 | -0.45001 | 9.21E-17 |
| CTSK | 0.794737 | 0.841968 | 0.083287 | 1.45E-10 | -0.34901 | 2.99E-10 |
| RP11-2C24.7 | 0.804851 | 0.516558 | -0.63979 | 1.46E-10 | -0.51383 | 3.75E-22 |
| ZNF549 | 0.246244 | 0.377572 | 0.616661 | 2.00E-10 | -0.79941 | 1.10E-69 |
| ZNF682 | 0.118321 | 0.234859 | 0.989095 | 2.08E-10 | -0.77442 | 8.49E-63 |
| CERS4 | 0.172511 | 0.235564 | 0.449429 | 2.15E-10 | -0.41963 | 1.44E-14 |
| ZNF461 | 0.057206 | 0.155783 | 1.445301 | 2.63E-10 | -0.48758 | 8.49E-20 |
| RP11-478C6.1 | 0.099056 | 0.211386 | 1.093559 | 2.68E-10 | -0.38414 | 2.87E-12 |
| FGF10 | 0.31078 | 0.421517 | 0.439697 | 3.06E-10 | -0.3245 | 5.53E-09 |
| ANKRD18B | 0.11298 | 0.340332 | 1.590878 | 3.30E-10 | -0.42934 | 3.03E-15 |
| LINC01535 | 0.38579 | 0.482351 | 0.322268 | 8.03E-10 | -0.50729 | 1.51E-21 |
| ZNF287 | 0.139881 | 0.248993 | 0.831903 | 1.02E-09 | -0.65712 | 1.89E-39 |
| MAATS1 | 0.122277 | 0.259846 | 1.0875 | 1.13E-09 | -0.53781 | 1.73E-24 |
| SLC43A3 | 0.18962 | 0.338473 | 0.835932 | 1.19E-09 | -0.75633 | 2.43E-58 |
| PRMT6 | 0.160457 | 0.212928 | 0.408183 | 1.35E-09 | -0.63908 | 9.31E-37 |
| NME5 | 0.099376 | 0.242065 | 1.284428 | 1.56E-09 | -0.61561 | 1.63E-33 |
| NUDT16P1 | 0.176119 | 0.141383 | -0.31695 | 2.56E-09 | -0.46752 | 3.94E-18 |
| AC005498.3 | 0.09552 | 0.348922 | 1.869026 | 2.77E-09 | -0.5919 | 1.68E-30 |
| ARHGDIB | 0.534596 | 0.404535 | -0.40218 | 3.83E-09 | -0.53361 | 4.58E-24 |
| AREG | 0.443604 | 0.330326 | -0.42538 | 8.14E-09 | -0.54895 | 1.23E-25 |
| RP11-474D1.2 | 0.550787 | 0.688875 | 0.322749 | 8.34E-09 | -0.47036 | 2.32E-18 |
| MAGEH1 | 0.30509 | 0.563327 | 0.884739 | 8.44E-09 | -0.52848 | 1.48E-23 |
| RPL39L | 0.30908 | 0.433942 | 0.489519 | 8.49E-09 | -0.74222 | 3.95E-55 |
| CTD-2535L24.2 | 0.502964 | 0.424423 | -0.24495 | 9.49E-09 | -0.37631 | 8.49E-12 |
| PIGR | 0.47416 | 0.632297 | 0.415227 | 1.01E-08 | -0.71419 | 2.48E-49 |
| ZSCAN12 | 0.322518 | 0.419698 | 0.379971 | 1.09E-08 | -0.36063 | 6.84E-11 |
| ZNF350 | 0.175398 | 0.283517 | 0.692802 | 1.09E-08 | -0.77134 | 5.22E-62 |
| ARMCX1 | 0.463034 | 0.571079 | 0.302572 | 2.11E-08 | -0.41449 | 3.23E-14 |
| KLHL34 | 0.423396 | 0.586921 | 0.471157 | 2.73E-08 | -0.65585 | 2.97E-39 |
| FAM72B | 0.064606 | 0.30037 | 2.216999 | 2.75E-08 | -0.71106 | 9.98E-49 |
| PKIA | 0.266232 | 0.365263 | 0.456251 | 2.79E-08 | -0.52281 | 5.26E-23 |
| FZD10 | 0.270302 | 0.419071 | 0.632625 | 2.88E-08 | -0.70541 | 1.17E-47 |
| ARSJ | 0.20508 | 0.18012 | -0.18723 | 3.23E-08 | -0.48967 | 5.60E-20 |
| FIRRE | 0.558481 | 0.368382 | -0.6003 | 3.28E-08 | -0.59934 | 2.03E-31 |
| SLFN11 | 0.168085 | 0.311176 | 0.888546 | 3.94E-08 | -0.40347 | 1.74E-13 |
| PVRL1 | 0.567796 | 0.59653 | 0.071221 | 4.29E-08 | -0.30758 | 3.58E-08 |
| SLFN12 | 0.31604 | 0.408856 | 0.371488 | 5.17E-08 | -0.49109 | 4.22E-20 |
| NRSN2 | 0.150681 | 0.217646 | 0.530486 | 5.76E-08 | -0.59786 | 3.09E-31 |
| RP5-858L17.1 | 0.146538 | 0.233282 | 0.670805 | 8.17E-08 | -0.54264 | 5.56E-25 |
| AMT | 0.450631 | 0.55351 | 0.296664 | 1.00E-07 | -0.64863 | 3.68E-38 |
| FBXO17 | 0.106373 | 0.18776 | 0.819762 | 1.52E-07 | -0.37713 | 7.58E-12 |
| RP11-114G11.4 | 0.653653 | 0.738229 | 0.175543 | 1.65E-07 | -0.41162 | 5.04E-14 |
| ZNF529 | 0.140179 | 0.257566 | 0.87767 | 1.81E-07 | -0.78174 | 1.02E-64 |
| SERP2 | 0.484363 | 0.602111 | 0.313941 | 1.89E-07 | -0.79458 | 2.81E-68 |
| LTB | 0.260341 | 0.317326 | 0.285563 | 2.03E-07 | -0.37174 | 1.58E-11 |
| ZNF329 | 0.304596 | 0.379194 | 0.31604 | 2.13E-07 | -0.67414 | 3.65E-42 |
| LINC00865 | 0.250344 | 0.319761 | 0.353082 | 4.81E-07 | -0.64936 | 2.86E-38 |
| HYDIN | 0.417702 | 0.494924 | 0.244735 | 5.47E-07 | -0.50443 | 2.76E-21 |
| AOX1 | 0.368424 | 0.505222 | 0.455552 | 6.02E-07 | -0.37074 | 1.80E-11 |
| GOLGA8A | 0.377174 | 0.457952 | 0.279967 | 7.29E-07 | -0.40419 | 1.56E-13 |
| RP11-267A15.3 | 0.307518 | 0.240577 | -0.35417 | 1.15E-06 | -0.47996 | 3.76E-19 |
| ZNF264 | 0.271561 | 0.348485 | 0.359819 | 1.24E-06 | -0.7529 | 1.54E-57 |
| ZNF300 | 0.432921 | 0.549485 | 0.343977 | 1.43E-06 | -0.78891 | 1.12E-66 |
| DTX3 | 0.249105 | 0.412336 | 0.727065 | 1.48E-06 | -0.50627 | 1.88E-21 |
| CIART | 0.282232 | 0.429786 | 0.606737 | 1.75E-06 | -0.43114 | 2.26E-15 |
| LINC01558 | 0.360243 | 0.302779 | -0.25071 | 1.80E-06 | -0.61703 | 1.06E-33 |
| ZNF550 | 0.250245 | 0.333396 | 0.413893 | 2.34E-06 | -0.73016 | 1.51E-52 |
| TMEM220 | 0.366112 | 0.468942 | 0.357123 | 2.38E-06 | -0.66613 | 7.29E-41 |
| NXPE4 | 0.475294 | 0.589238 | 0.31003 | 2.83E-06 | -0.38953 | 1.34E-12 |
| CTD-2245F17.3 | 0.048921 | 0.212192 | 2.116857 | 3.03E-06 | -0.71061 | 1.21E-48 |
| B3GALNT1 | 0.255659 | 0.39372 | 0.622948 | 3.46E-06 | -0.56661 | 1.49E-27 |
| DAPP1 | 0.548969 | 0.438383 | -0.32453 | 4.22E-06 | -0.78976 | 6.49E-67 |
| REP15 | 0.32443 | 0.462981 | 0.513045 | 4.62E-06 | -0.45628 | 3.04E-17 |
| RP11-543D5.1 | 0.78395 | 0.66281 | -0.24217 | 5.27E-06 | -0.58587 | 8.93E-30 |
| ZNF718 | 0.257157 | 0.362624 | 0.495825 | 5.32E-06 | -0.69966 | 1.36E-46 |
| ZNF844 | 0.10758 | 0.177828 | 0.725066 | 5.54E-06 | -0.74768 | 2.40E-56 |
| KRT20 | 0.627403 | 0.490667 | -0.35465 | 6.94E-06 | -0.66199 | 3.31E-40 |
| CYP2B7P | 0.569233 | 0.664359 | 0.222943 | 8.14E-06 | -0.40341 | 1.75E-13 |
| ZNF83 | 0.186336 | 0.25263 | 0.439116 | 9.69E-06 | -0.64573 | 9.96E-38 |
| FUZ | 0.11894 | 0.187602 | 0.65744 | 1.10E-05 | -0.69175 | 3.58E-45 |
| ZNF254 | 0.213807 | 0.355072 | 0.7318 | 1.37E-05 | -0.40571 | 1.24E-13 |
| SLC14A1 | 0.826394 | 0.85258 | 0.045006 | 1.54E-05 | -0.36411 | 4.34E-11 |
| CTC-260E6.6 | 0.150351 | 0.25625 | 0.769223 | 2.45E-05 | -0.54681 | 2.05E-25 |
| ITPRIPL1 | 0.234572 | 0.310376 | 0.403981 | 2.46E-05 | -0.48074 | 3.23E-19 |
| ZNF345 | 0.124742 | 0.213975 | 0.778499 | 2.62E-05 | -0.75063 | 5.11E-57 |
| RP1-122P22.2 | 0.192964 | 0.268867 | 0.478558 | 4.01E-05 | -0.36084 | 6.65E-11 |
| RP11-680F8.1 | 0.402575 | 0.460171 | 0.192913 | 4.49E-05 | -0.52392 | 4.11E-23 |
| BEX4 | 0.238163 | 0.383835 | 0.688541 | 4.66E-05 | -0.42213 | 9.70E-15 |
| VWDE | 0.210554 | 0.290819 | 0.465933 | 6.48E-05 | -0.60303 | 6.94E-32 |
| TRIM29 | 0.584584 | 0.530157 | -0.14099 | 6.72E-05 | -0.60973 | 9.63E-33 |
| LINC00605 | 0.238414 | 0.335979 | 0.494902 | 0.000107 | -0.59758 | 3.35E-31 |
| RP11-255H23.2 | 0.228214 | 0.319931 | 0.487371 | 0.000121 | -0.74959 | 8.85E-57 |
| RP11-47P18.1 | 0.386612 | 0.331594 | -0.22147 | 0.000132 | -0.58781 | 5.23E-30 |
| LRRC34 | 0.042743 | 0.10214 | 1.256806 | 0.000135 | -0.41803 | 1.86E-14 |
| SHC3 | 0.324214 | 0.41733 | 0.364243 | 0.000138 | -0.47513 | 9.46E-19 |
| SMOC1 | 0.306526 | 0.369637 | 0.270101 | 0.000157 | -0.52199 | 6.31E-23 |
| HOXB-AS1 | 0.509498 | 0.569888 | 0.161603 | 0.000189 | -0.48011 | 3.65E-19 |
| SATB2 | 0.211132 | 0.278901 | 0.401606 | 0.000194 | -0.47718 | 6.41E-19 |
| GSTM1 | 0.283873 | 0.388053 | 0.45101 | 0.000266 | -0.56081 | 6.53E-27 |
| HOXB2 | 0.59589 | 0.654225 | 0.134742 | 0.000278 | -0.69942 | 1.50E-46 |
| LINC01214 | 0.65021 | 0.737442 | 0.181622 | 0.000358 | -0.47685 | 6.83E-19 |
| HSPA1A | 0.096764 | 0.279938 | 1.532558 | 0.000359 | -0.70881 | 2.68E-48 |
| RP11-370I10.12 | 0.285676 | 0.43708 | 0.613519 | 0.000361 | -0.70091 | 8.01E-47 |
| TMEM106A | 0.181997 | 0.377629 | 1.053051 | 0.000371 | -0.70028 | 1.04E-46 |
| PON3 | 0.237874 | 0.218562 | -0.12215 | 0.000391 | -0.50662 | 1.74E-21 |
| PSMA8 | 0.653496 | 0.682647 | 0.062961 | 0.00041 | -0.31883 | 1.05E-08 |
| ZNF583 | 0.067228 | 0.116099 | 0.788217 | 0.000436 | -0.62681 | 5.00E-35 |
| FAM179B | 0.071508 | 0.14124 | 0.981959 | 0.000455 | -0.4644 | 7.01E-18 |
| FAM127A | 0.230652 | 0.38869 | 0.752901 | 0.000501 | -0.61007 | 8.68E-33 |
| AHR | 0.737258 | 0.779158 | 0.079747 | 0.000512 | -0.40488 | 1.41E-13 |
| LYZ | 0.654523 | 0.546993 | -0.25892 | 0.000609 | -0.62075 | 3.36E-34 |
| LY6G6E | 0.66173 | 0.514478 | -0.36313 | 0.000941 | -0.68342 | 1.01E-43 |
| BHLHB9 | 0.374776 | 0.468146 | 0.320929 | 0.000949 | -0.36937 | 2.17E-11 |
| GNG4 | 0.296964 | 0.28615 | -0.05352 | 0.001055 | -0.7362 | 8.03E-54 |
| PHLDA3 | 0.311923 | 0.361556 | 0.213027 | 0.001243 | -0.46736 | 4.06E-18 |
| ZNF879 | 0.198436 | 0.300889 | 0.600563 | 0.001297 | -0.7344 | 1.94E-53 |
| RAB32 | 0.162298 | 0.161309 | -0.00882 | 0.001433 | -0.58531 | 1.04E-29 |
| RP1-97D16.1 | 0.340377 | 0.418368 | 0.297641 | 0.001577 | -0.54444 | 3.62E-25 |
| LINC01413 | 0.547083 | 0.57044 | 0.060316 | 0.001671 | -0.39644 | 4.92E-13 |
| CROT | 0.104513 | 0.120442 | 0.204649 | 0.002123 | -0.53686 | 2.16E-24 |
| RP11-616M22.7 | 0.190825 | 0.262663 | 0.460964 | 0.002429 | -0.39042 | 1.18E-12 |
| NINL | 0.275117 | 0.359369 | 0.385417 | 0.002439 | -0.54136 | 7.52E-25 |
| BST2 | 0.54068 | 0.433541 | -0.31861 | 0.002614 | -0.76001 | 3.23E-59 |
| RP3-407E4.4 | 0.552478 | 0.569934 | 0.044877 | 0.003252 | -0.49486 | 1.98E-20 |
| MPC2 | 0.840457 | 0.862552 | 0.037437 | 0.00432 | -0.34567 | 4.52E-10 |
| ZNF614 | 0.039332 | 0.11527 | 1.551233 | 0.004337 | -0.65599 | 2.83E-39 |
| RP4-584D14.6 | 0.374822 | 0.446369 | 0.252032 | 0.004771 | -0.38693 | 1.94E-12 |
| MAP10 | 0.330773 | 0.406167 | 0.296231 | 0.004924 | -0.78476 | 1.55E-65 |
| NT5DC4 | 0.880631 | 0.81152 | -0.11791 | 0.005183 | -0.67028 | 1.56E-41 |
| ZNF518B | 0.382973 | 0.461699 | 0.269709 | 0.005456 | -0.79185 | 1.69E-67 |
| SLC2A10 | 0.061719 | 0.119309 | 0.95091 | 0.006431 | -0.4971 | 1.25E-20 |
| LY6G6D | 0.489753 | 0.399914 | -0.29236 | 0.007228 | -0.83578 | 1.15E-81 |
| LPL | 0.360321 | 0.421292 | 0.225539 | 0.00847 | -0.40146 | 2.34E-13 |
| QPCT | 0.336622 | 0.33584 | -0.00336 | 0.008974 | -0.54528 | 2.96E-25 |
| RGN | 0.599592 | 0.667641 | 0.155092 | 0.009726 | -0.65901 | 9.63E-40 |
| SPDYC | 0.7285 | 0.736739 | 0.016225 | 0.010579 | -0.30285 | 5.92E-08 |
| ZNF543 | 0.11813 | 0.30725 | 1.379042 | 0.014701 | -0.78235 | 6.96E-65 |
| AK4 | 0.234437 | 0.25265 | 0.107942 | 0.014756 | -0.46269 | 9.58E-18 |
| TUSC1 | 0.110269 | 0.105212 | -0.06773 | 0.017586 | -0.55288 | 4.70E-26 |
| TRPV2 | 0.660209 | 0.69524 | 0.07459 | 0.018387 | -0.39013 | 1.23E-12 |
| CTD-2319I12.4 | 0.25428 | 0.341927 | 0.427268 | 0.019438 | -0.58627 | 8.00E-30 |
| SP5 | 0.381379 | 0.417231 | 0.129619 | 0.026453 | -0.39702 | 4.52E-13 |
| RP11-357H14.17 | 0.229308 | 0.21336 | -0.104 | 0.03047 | -0.40829 | 8.39E-14 |
| SEPHS2 | 0.205682 | 0.18588 | -0.14605 | 0.041166 | -0.47188 | 1.75E-18 |
| FGD1 | 0.231617 | 0.292957 | 0.338945 | 0.047425 | -0.38628 | 2.12E-12 |
