# Supplementary material for: A methylation‐driven gene panel predicts survival in patients with colon cancer
Source: FEBS Open Bio. 2021 Jul 28;11(9):2490–506. doi: 10.1002/2211-5463.13242 (PMC8409306; doi:10.1002/2211-5463.13242)
Supplement: Supplementary file 4 — Table S3. Clinical characteristics of 281 colon cancer patients included in survival analysis. [file FEB4-11-2490-s001.docx]

**Table S3.** Clinical characteristics of 281 colon cancer patients included in survival analysis.

| Variable | Number of cases (n = 281) |
| --- | --- |
| Age (years) |  |
| <60/>=60 | 96/185 |
| Gender |  |
| Female/Male | 128/153 |
| History of colon polyps |  |
| No/Yes/NA | 163/50/68 |
| Pretreatment CEA level (ng/μl) |  |
| <5.0/>=5.0/NA | 123/61/97 |
| T stage |  |
| T1/T2/T3/T4 | 8/42/194/37 |
| N stage |  |
| N0/N1/N2 | 162/73/46 |
| M stage |  |
| M0/M1/Mx | 192/40/49 |
| TNM stage |  |
| Ⅰ/Ⅱ/Ⅲ/Ⅳ/NA | 43/106/82/40/10 |
| Venous invasion |  |
| No/Yes/NA | 185/58/38 |
| Tumor location |  |
| Right colon/Left colon/NA | 164/98/19 |

CEA, carcinoembryonic antigen; NA, not available.
