# Supplementary material for: A methylation‐driven gene panel predicts survival in patients with colon cancer
Source: FEBS Open Bio. 2021 Jul 28;11(9):2490–506. doi: 10.1002/2211-5463.13242 (PMC8409306; doi:10.1002/2211-5463.13242)
Supplement: Supplementary file 5 — Table S4. 12 methylation‐driven genes significantly associated with overall survival of colon cancer patients screened by univariate Cox regression analysis in the training set (n = 141). [file FEB4-11-2490-s003.docx]

**Table S4.** 12 methylation-driven genes significantly associated with overall survival of colon cancer patients screened by univariate Cox regression analysis in the training set (n=141).

| Gene | HR (95% CI) | P value |
| --- | --- | --- |
| HOXB2 | 0.109 (0.019 - 0.615) | 0.012 |
| FGD1 | 0.005 (0.000 - 0.327) | 0.013 |
| AC009014.3 | 0.001 (0.000 - 0.213) | 0.014 |
| CD40 | 0.046 (0.004 - 0.567) | 0.016 |
| TMEM88 | 0.007 (0.000 - 0.536) | 0.025 |
| AMT | 0.008 (0.000 - 0.634) | 0.031 |
| SERP2 | 0.016 (0.000 - 0.703) | 0.032 |
| ZNF345 | 0.005 (0.000 - 0.730) | 0.037 |
| ARHGDIB | 0.024 (0.001 - 0.813) | 0.038 |
| FAM179B | 0.002 (0.000 - 0.835) | 0.044 |
| PRMT6 | 0.000 (0.000 - 0.849) | 0.046 |
| TMEM35 | 0.010 (0.000 - 0.947) | 0.047 |

HR, hazard ratio; CI, confidence interval.
